# Spray-Drying of Electrode Materials for Lithium- and Sodium-Ion Batteries

**DOI:** 10.3390/ma11071076

**Published:** 2018-06-25

**Authors:** Benedicte Vertruyen, Nicolas Eshraghi, Caroline Piffet, Jerome Bodart, Abdelfattah Mahmoud, Frederic Boschini

**Affiliations:** GREENMAT, CESAM Research Unit, University of Liege, Chemistry Institute B6, Quartier Agora, Allée du 6 août, 13, B-4000 Liege, Belgium; neshraghi@uliege.be (N.E.); caroline.piffet@uliege.be (C.P.); jerome.bodart@uliege.be (J.B.); abdelfattah.mahmoud@uliege.be (A.M.); frederic.boschini@uliege.be (F.B.)

**Keywords:** spray-drying, batteries, lithium ion batteries, sodium ion batteries, electrode materials, solution synthesis, suspensions

## Abstract

The performance of electrode materials in lithium-ion (Li-ion), sodium-ion (Na-ion) and related batteries depends not only on their chemical composition but also on their microstructure. The choice of a synthesis method is therefore of paramount importance. Amongst the wide variety of synthesis or shaping routes reported for an ever-increasing panel of compositions, spray-drying stands out as a versatile tool offering demonstrated potential for up-scaling to industrial quantities. In this review, we provide an overview of the rapidly increasing literature including both spray-drying of solutions and spray-drying of suspensions. We focus, in particular, on the chemical aspects of the formulation of the solution/suspension to be spray-dried. We also consider the post-processing of the spray-dried precursors and the resulting morphologies of granules. The review references more than 300 publications in tables where entries are listed based on final compound composition, starting materials, sources of carbon etc.

## 1. Introduction

Secondary batteries such as Li-ion, Na-ion, or related batteries are complex electrochemical devices [1,2]. Their optimal performance relies on the harmonious operation of all parts, which depends not only on the individual characteristics of the positive electrode (cathode), the negative electrode (anode) and the electrolyte, but also on the interfaces between them. It is well known that microstructure effects have a strong impact on properties as can be illustrated by the case of the electrodes. On the one hand, the composition of the active electrode material determines electrode voltage and theoretical capacity. On the other hand, the microstructure (both of the active material component and of the composite electrode as a whole) strongly influences the actual electrochemical performance at high charge-discharge rates (rate capability). The microstructure also determines the specific surface area in contact with the electrolyte, with effects on kinetics and cycling stability. Finally, the microstructure has an influence on the packing efficiency and therefore on the energy density (=energy per unit of volume) of the battery.

**Figure 1:**
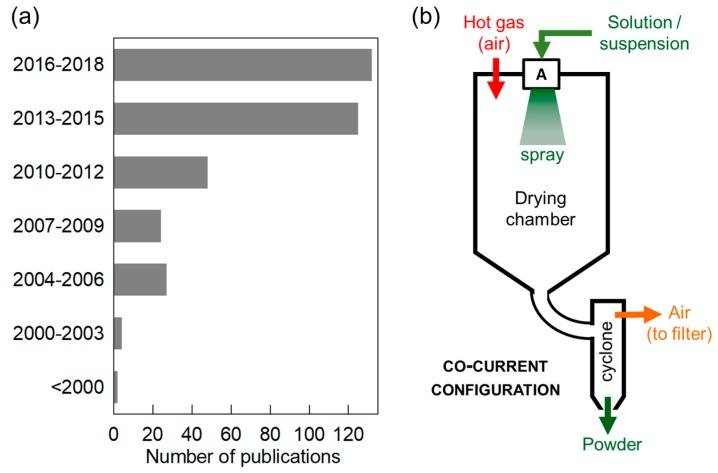
(**a**) Number of publications related to spray-drying of electrode materials for Li-ion, Na-ion and related batteries; (**b**) Schematic of a spray-dryer, showing the case of a co-current configuration and bi-fluid nozzle atomization.

This key role of the microstructure means that the selection of a synthesis and/or shaping method can have a decisive impact on practical performance indicators. As a result, the literature on the synthesis of electrode materials has been increasing at a tremendous rate, with reports of a wide variety of routes for each active electrode material candidate. Searching for the most appropriate preparation procedure(s) in each particular case is a legitimate and sound objective. However, the possibility to transfer results from the laboratory scale of typically a few grams to industrially relevant production conditions should be taken into account from an early stage. This is especially important in the case of electrode materials, since the microstructure is often one of the most impacted characteristics in case of upscaling, due to heat-transfer issues when going from small volumes to larger batches or continuous production. Comparatively easy upscaling is one of the strengths of spray-drying [3], a versatile and robust technique whose classical fields of applications (in the food and pharmaceutical industries) have recently been expanding to include the synthesis/shaping of electrode materials (Figure 1a).

**Figure 2:**
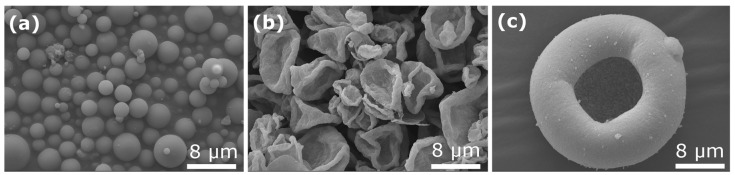
Examples of morphology of as-sprayed granules: (**a**) precursor of Na_3_V_2_(PO_4_)_2_F_3_, spray-drying of aqueous solution, bi-fluid nozzle atomization; (**b**) same as (**a**) with addition of carbon nanotubes in the solution; (**c**) silicon, spray-drying of suspension in alcohol, fountain mode. All three micrographs are unpublished scanning electron microscope (SEM) micrographs from the authors’ own work.

In a spray-dryer (Figure 1b), a solution or suspension is sprayed into droplets and the solvent or liquid in each droplet is evaporated by a hot gas flow (usually air), resulting in a dry powder (see Figure 2 for a few examples of granule morphologies). Larger quantities can be obtained simply by spraying a larger volume over a longer time, without modification of the conditions experienced by each individual droplet. Several experimental configurations exist, as briefly discussed in Section 2.

**Figure 3:**
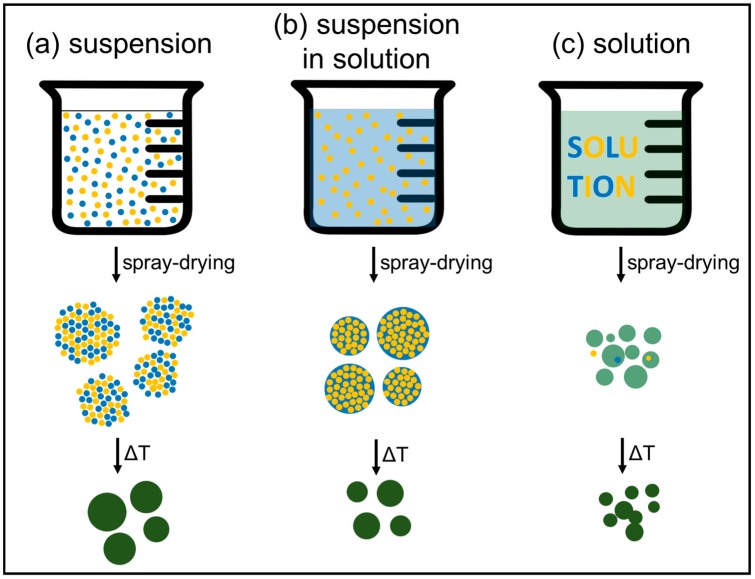
Spray-drying of (**a**) a suspension of solid particles (blue and yellow) dispersed in a non-solvent (transparent); (**b**) a suspension of solid particles (yellow) in a solution (light blue); (**c**) a solution (light green) of soluble precursors. All schematics consider the case where the spray-dried precursor is further transformed into the final phase (dark green) by heat treatment.

Spray-drying can be applied to suspensions (Figure 3a) or solutions (Figure 3c) but also to the intermediate case of suspensions in solutions (Figure 3b). In all of these cases it can be used as a shaping technique, typically to obtain spherical granules. This application of spray-drying is commonly encountered in the food and pharmaceutical industries, and to granulate nanopowders into re-dispersible micrometric granules for safer handling and transport. In the context of electrode materials, this version of spray-drying (i.e., without post-processing heat treatment) is usually applied to suspensions containing both small particles of active material and some form of solid conducting carbon. The objective is then to achieve a good mixing of active material and carbon and to obtain granules with good flowability and packing properties for efficient electrode formulation.

As depicted in Figure 3, spray-drying can also be used to intimately mix reactants in view of ulterior transformation into the final product by heat treatment. This version of spray-drying is the most common in the field of electrode materials, as will be seen in this review. Mixing of the reactants can occur at the atomic scale when starting from a solution, whereas homogeneity is determined by the (nano)particle size when starting from a suspension or a suspension in a solution. In spray-drying, the objective is the evaporation of the droplet liquid, and decomposition of the solid is not supposed to happen at this stage (especially in the case of heat sensitive pharmaceuticals or food). If further heat treatment is needed to form the final compound, partial decomposition during spray-drying is obviously not a problem. The technique of spray pyrolysis for powder synthesis targets decomposition and requires much higher temperatures, which are reached by spraying into a tubular furnace setup or in a flame. Spray pyrolysis will not be discussed here (see [4,5,6,7,8] for a few examples).

The present review is focused on spray-drying for electrode materials (see Table 1) and is to our knowledge the first of its kind. Readers interested in a more general overview of the technique and its broad-ranging scope of applications can refer to reviews such as those by Nandiyanto and Okuyama [9] (on particle sizes and morphologies), Mezhericher et al. [10] (on models of droplet drying), Zbicinski [11] (on modeling of industrial spray-dryers), Stunda-Zujeva et al. [3] (on spray-drying for ceramics), Deshmukh et al. [12] and Singh et al. [13] (on spray-drying for drug delivery), Gharsallaoui et al. [14] (on microencapsulation of food ingredients), Schuck et al. [15] (on spray-drying for the dairy industry) and references therein.

This review deals primarily with chemistry- and microstructure-related topics such as the formulation of solutions and suspensions, the impact of spray-drying parameter selection, or strategies to create composites with conducting carbon. It should be seen as a complement to available reviews that focus on the discussion and benchmarking of electrochemical performance in materials based on the same (family of) compound(s) or intended for one type of battery/electrode (see for examples [2,16,17,18,19]), where much less attention is paid to the details of the synthesis procedures.

## 2. Experimental Parameters in Spray-drying

Spray-dryers exist in all sizes, from table-top systems to industrial production units. In the primary scientific literature, the most common systems are home-made equipment, commercial table-top systems [368,369] and commercial (small) pilot-scale systems. As an example, our group started working on spray-drying at the beginning of the 2000s with a table-top Buchi Mini Spray-dryer B-190 (Büchi Labortechnik AG, Switzerland) and now owns two Mobile Minor^TM^ units, which can evaporate up to 5.5 kg H_2_O per hour and correspond to the smallest-but-one R&D systems on the catalogue of a provider of industrial spray-drying technology (GEA). One of the largest-scale tests for electrode materials (in scientific publications) was reported by Han et al. [221] for the synthesis of 15 kg Li_4_Ti_5_O_12_.

Basically, all spray-dryers include (i) an atomizer (most often a bi-fluid nozzle or a rotating wheel) where the liquid feedstock is sprayed into droplets; (ii) a drying chamber where a hot gas flow (injected in co-current or counter-current configuration) evaporates the liquid and (iii) a final section where the dry powder is separated from the gas flow and collected, sometimes at several collection points depending on particle size. A typical configuration is schematized in Figure 1b. Ancillary equipment can be added to filter the outgoing gas, to carry out spray-drying using an inert gas instead of air or to condense vapors of organic solvents when non-aqueous solutions/suspensions are used. In this latter case, systems specially designed to prevent explosion/fire risks should be used.

When reporting on spray-drying experiments, good practice would be to provide information not only about the composition of the liquid feedstock but also about the spray-drying setup and experimental parameters such as inlet temperature, outlet temperature, and feedstock flow rate. When commercial equipment is used, additional parameters (such as air/gas pressure of the bifluid nozzle or rotating atomizer, etc.) should also be reported. A recent review by Arpagaus et al. [368] includes a section about electrode materials, focusing on a few publications where detailed spray-drying parameters are provided together with data on particle morphology and electrochemical performance. In most papers, however, information on the spray-drying parameters is missing or incomplete as illustrated by Table A1 in Appendix A for the case of layered oxide compounds.

Some of these parameters (for example the inlet temperature or the flow rate) can be selected independently but others, notably the outlet temperature, are the consequence of the selected parameters. Typically, increasing the inlet temperature or decreasing the feedstock flow rate results in an increase of the outlet temperature. In practice, the ‘selectable’ parameters are often adjusted to target a specific outlet temperature. Indeed, due to the wet-bulb effect [370], the outlet temperature is often the highest temperature experienced by the material in the spray-dryer (at least in the most common co-current configuration). The outlet temperature, therefore, determines to a large extent how dry the final powder will be and must be carefully controlled especially when spray-drying heat sensitive compounds.

## 3. Formulation of Solutions/Suspensions: Inorganic Components

As shown in Table 1, electrode materials prepared by spray-drying span a broad range of compositions, from elements to oxides, phosphates, sulfides, fluorides, and others. In most cases, the spray-drying step results in the formation of a precursor, which will be transformed into the final phase through ulterior treatment (most commonly through heat treatment). This section describes and discusses the formulation of solutions or suspensions used as feedstock for spray-drying. Some specific cases are taken as illustrative examples. More systematic information is provided in Table A2 in Appendix A, which consists of an inventory of the starting materials used in the publications referenced in this review.

### 3.1. Solvent/Liquid Phase

The most common solvent (for solutions) or liquid medium (for suspensions) is water. This is easily explained by considering that water is cheap, safe and non-toxic. As shown in Table 2, alcohols are also used, either pure or mixed with water. Other liquids are much less common (see Table 2). From the physico-chemical point of view, the two most important selection criteria are the vaporization temperature of the liquid (which must be in the adequate range for the spray-drying equipment) and its solvent/non-solvent character with respect to the reactants. However, safety, recycling, and prevention of release in the atmosphere must be addressed when using organic liquids, typically through appropriate equipment (fire/explosion-proof equipment, condensing of solvent vapors, etc.).

### 3.2. Solutions

The discussion in this section and the next is illustrated with the case of the AMO_2_ layered oxide compounds (A = Li^+^, Na^+^; M = one or several of Li, Ni, Mn, Co, Al, …). The references in Table 3 are sorted into categories labeled A to H according to the type of solution/suspension.

An essential point to consider when preparing a solution for spray-drying is that, except volatiles, all components will be present in the spray-dried powder. This restricts the choice of counter-ions and of all additives to compounds that will be decomposed during ulterior heat treatment, or do not interfere with functionality. With this in mind, aqueous solutions can be prepared (1) by adding soluble salts in water or (2) by dissolving less soluble but cheaper precursors.

In the first case, nitrates and acetates (for cations) or ammonium salts (for anions) are common choices due to their low decomposition temperatures. This is illustrated by Category A in Table 3 where acetates and/or nitrates were selected as water-soluble salts of Ni, Co, Mn or Al. Regarding ammonium salts as a source of anions, the most common example is probably (NH_4_)H_2_PO_4_ which is a popular precursor in the synthesis of phosphates (see Table A2 in Appendix A).

In the second case, dissolution in (aqueous) acid is the most frequent. Since hard acids (such as HNO_3_) usually drive the pH to very low values which might damage the spray-drying equipment, dissolution in milder acids such as citric or acetic acids (or, more imaginatively, polyacrylic acid [159]) is often preferred when possible (see Categories B and D in Table 3). The pH can also be brought back to less acidic values by addition of bases that do not introduce foreign cations, such as ammonia solution. Incidentally, the possibility of auto-combustion occurring during the early stages of the heat treatment of the spray-dried material should be kept in mind when nitrates and organics are simultaneously present. The probability is enhanced if ammonium nitrate has been formed by neutralizing an excess of nitric acid by ammonia solution.

In many cases, complexation of the metallic cations may be needed, either to prevent precipitation of a less soluble salt when different soluble salts are mixed in solution or if the solubility product of a metal hydroxide (less commonly a carbonate) is exceeded when adjusting the pH to more basic values.

Formation of stable complexes (such as citrates, see Category C in Table 3) is also a strategy to favor a homogeneous distribution of the chemical species in the spray-dried particles. This is relevant for cases of complex compositions where sequential precipitation might occur during the drying of the droplet, i.e., precipitation of several phases starting with the least soluble and going on to the most soluble. This raises the more general question of the extent to which the homogeneity of a solution can be retained in a spray-dried precursor. On the one hand, the actual impact of this issue is limited since, by comparison with other synthesis techniques, the degree of inhomogeneity is restricted by the small size of the droplets. On the other hand, maximum homogeneity remains desirable for ulterior formation of the target phase. This is a case-by-case issue since it depends on solubilities of specific compounds, however helpful guidelines could be achieved if more authors reported relevant data in their publications. Even if a detailed characterization of the homogeneity in the as-sprayed material is difficult to obtain, valuable insight might be gained by simpler procedures. One such procedure is to collect X-ray diffractograms on samples taken out of the furnace at lower temperatures during the heating ramp, in order to identify which phases form first.

**Figure 4:**
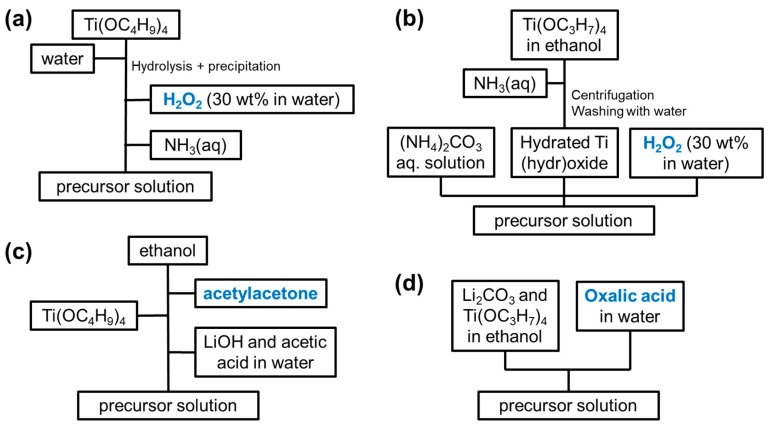
Procedures to prepare an aqueous solution starting from titanium alkoxide, as proposed by (**a**) [222,249]; (**b**) [127]; (**c**) [229,238]; (**d**) [243,248,251].

The above discussion focused on electrode materials (such as oxides or (fluoro)phosphates) for which soluble precursors are available. In the case of titanate or silicate electrode materials, the preparation of solutions is more difficult because few precursors are soluble in aqueous solutions of less-than-extreme pH. Chloride and/or alkoxide precursors (such as TEOS Si(OC_2_H_5_)_4_ [350,352], titanium isopropoxide Ti(OC_3_H_7_)_4_ [128,132,212,227,235,243,248,251] or tert-butoxide Ti(OC_4_H_9_)_4_ [211,222,229,238,239,240,241,249,250,265], …) can be solubilized in alcohol but hydrolysis takes place when mixing with water, leading to the precipitation of SiO_2_ or TiO_2_ unless special care is taken as in the strategies summarized in Figure 4.

When the composition for the solution has been settled, other parameters still need to be decided on. One of them is the concentration of the solution. A naïve view is that it should be as high as possible, in order to minimize the amount of solvent to be evaporated. However, too high concentrations can lead to gel formation or precipitation in the atomization nozzle. Besides, the solution concentration influences the morphology of spray-dried particles. All other parameters being equal (esp. the inlet temperature and the droplet size), a higher concentration means that the solubility limit is exceeded sooner during vaporization of the solvent and crust formation, therefore, occurs at a larger droplet diameter (collapse or cracking may take place later if the mechanical strength of the crust is too low). The concentration of the solution is, therefore, best adjusted in conjunction with other parameters (inlet temperature, flow rate, atomization parameters) in order to optimize the temperature profile of the drying process as a function of the priorities. Examples of such priorities can be a specific type of morphology but also the avoidance of partial decomposition or the minimization of residual humidity. This last point is important in relation to the possible post-spray-drying aging of the spray-dried material. Generally speaking, it is not recommended to store as-sprayed materials if they are made up of hygroscopic compounds (such as nitrates or, to a lesser extent, acetates) or if inorganic condensation mechanisms can take place (typically for Ti- or Si-based precursors of oxides). In such cases, the as-sprayed materials should be heat-treated to a temperature selected to obtain a stable (and, therefore, reproducible) intermediate state of the material.

### 3.3. Suspensions

Here we consider as a suspension all cases where at least one component is insoluble or only partially soluble in the liquid medium (=cases a and b in Figure 3). Spray-drying of suspensions can be used to mix reactants before heat treatment (Categories E and F in Table 3), to mix an active material with conductive carbon (Category G in Table 3), or both. It can also be used as a “shaping-only” method to prepare spherical granules of an active material (Category H in Table 3).

The first point made when discussing solutions is also valid for suspensions: except volatiles, everything that is added to the suspension will be present in the spray-dried powder. Therefore, oxides, carbonates, oxalates or hydroxides are common choices since they decompose during the heat treatment in air without leaving residues. In theory, this also applies to the selection of additives such as cationic dispersing agents, where ammonium counter-ions should be preferred to sodium counter-ions, although quantities remain low.

When considering spray-drying of suspensions, the stability of the suspension is obviously an important requirement. What is called a “stable” suspension in this context may however vary. At one end of the spectrum, the criterion may be that there is no visible sign of sedimentation when the suspension is under stirring and when it is pumped through tubes to the atomization head. At the other end, a stable suspension can be characterized by long-term stability and low aggregation thanks to efficient repulsion between individual particles. Whatever the case, a particle size of about 1 µm or below is always preferable. If the good mixing of small particles is retained in the spray-dried material, the small particle size is also favorable for the formation of the final phase since diffusion distances during heat treatment will be correspondingly short. Minimizing diffusion distances is also the reason why some suspensions involve pre-synthesized co-precipitates of several cations (e.g., (Co,Ni,Mn)(OH)_x_ [149,165,188], (Co,Ni,Mn)O_x_ [150], (Ni,Mn) oxalate [210], (Fe,Mn)_3_(PO_4_)_2_·xH_2_O [312,313,314,319]). If the coprecipitate is isolated by filtration or centrifugation before being redispersed into the suspension, its stoichiometry should be checked and the possibility of partial redissolution in suspension should be kept in mind.

Decreasing the particle size can be achieved by ball-milling a suspension of the larger particles in a liquid medium. An advantage of spray-drying is that the ball-milled suspension can often be used directly as feedstock for spray-drying if the liquid medium is suitable (see [143,216,275,283] for a few examples). Another possibility is to use commercial nanopowders to prepare the suspension. It should be noted that the viscosity of suspensions of very small particles (typically below about 100 nm) increases rapidly with solid loading. Also, depending on the fabrication process and/or aging on storage, the surface of the nanoparticles may be chemically different from the core (e.g., hydroxyl-rich or carbonated surface of some oxide particles), which might strongly affect their dispersion behavior and should also be taken into account when calculating stoichiometric proportions in multi-component suspensions. Finally, the high surface area of nanopowders means that they will be particularly affected if surface reaction or partial dissolution of the particle can occur in the liquid medium. These effects are rarely spectacular but should be considered when adjusting pH or when an unexpected behavior needs explaining.

As briefly mentioned above, the formulation of a suspension may involve the addition of a dispersing agent, which may be cationic, neutral or anionic and acts through electrostatic and/or steric effects. The formulation of suspensions of several powders (multicomponent suspensions) with long-term stability often becomes a formidable task, made even more complicated if the solid powders are suspended in a solution instead of in a simple liquid medium. At least in the context of electrode material synthesis, the formulation of multicomponent suspensions usually targets only practical stability, where “practical” means long enough for the spray-drying procedure.

In the case of suspensions prepared for spray-drying, other additives such as polyethylene glycol or polyvinyl alcohol may be added as binder to increase the cohesion and mechanical strength of the spray-dried granules. These binders usually tend to increase the viscosity of the suspension, which brings us back to the selection of the solid loading. Besides this practical limit associated with the maximum viscosity acceptable for the spray-drying equipment, the criteria for selecting solid loading are similar to those discussed for deciding the concentration of solutions: the solid loading in a suspension should be adjusted in conjunction with the primary spray-drying parameters (injection mode, inlet temperature, feed rate, atomization parameters) depending on the targeted size and morphology of granules. In the case of multicomponent suspensions, additional complexity is created if the different components have different particle sizes or in the case of suspension-in-solutions (Figure 3b), which may lead to distribution gradients in the dried granules. This phenomenon has not yet been studied in detail in the case of active electrode materials but other (simpler) systems have been investigated [372,373,374].

## 4. Formulation of Solutions/Suspensions: Organic/Carbon Components

This section focuses on organic (macro)molecules (listed in Table 4, with references) or carbon compounds (listed in Table 5, with references) which may be added to the solution/suspension for several reasons.

As already mentioned above, soluble organic (macro)molecules may function as complexing agents, dispersing agents, binders, etc. For example, carboxylic acids can be used as acids, as reducing agents or as complexing agents (especially when transformed into carboxylate ions by pH adjustment). Citric acid is an extremely popular choice, as can be seen in Table 4.

Another example is that of synthetic polymers which are used as dispersing agents, thickeners and/or binders. Their exact role is not always defined and depends in part on the molecular mass. Common choices are polyethylene glycol (PEG), polyvinylalcohol (PVA) and polyvinylpyrrolidone (PVP) (see Table 4). PEG, PVA and PVP are of the non-ionic (steric) type but cationic additives are also reported (ammonium polycarboxylate [216,221], sodium carboxymethylcellulose [68,70,119,227,333], sodium dodecyl benzene sulfonate (SDBS) [53,65]).

All these (macro)molecules and a whole range of other organic compounds (see Table 4) can also be used as precursors transforming into carbon during heat treatment in inert/reducing atmosphere. Indeed, a frequent concern when synthesizing electrode materials is that the (relatively) low intrinsic electronic conductivity of many active materials is a limit to the kinetics of the electrochemical reactions. In order to improve electron transport to the active material, common approaches are the formation of a coating and/or a composite with some form of conducting carbon.

Another reason for using composites with carbon is that some active materials (such as Si) undergo very large expansions/contractions on electrochemical cycling; in such cases carbon can be used as a buffer to limit the volume variations and the degradation of performance that results from loss of connectivity inside the electrode.

**Figure 5:**
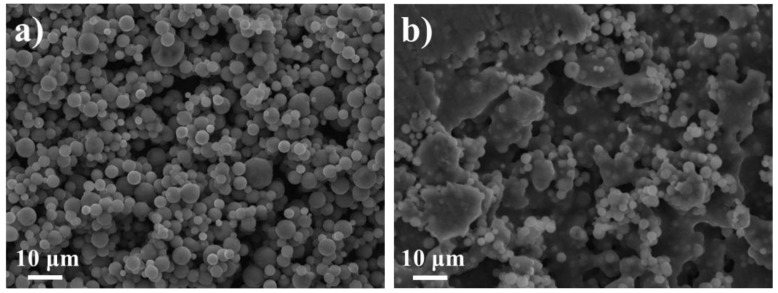
SEM images of as-sprayed powders after 6-h exposure to atmosphere: (**a**) the tin oxalate-dextrin composite is stable; (**b**) the tin oxalate-sucrose composite is hygroscopic. (Adapted from [122] with permission—© 2014 Wiley-VCH Verlag).

Since spray-drying usually yields relatively large particles (a few microns to a few tens of microns), surface coating of the spray-dried particles is not good enough for compounds that require intimate mixing with carbon. One possibility is to grind the spray-dried particles and mix them with carbon. Another approach is to include carbon or a carbon precursor in the spray-drying feedstock solution/suspension. Citric acid and saccharides such as glucose or sucrose are amongst the most common soluble carbon precursors (see Table 4), transforming into more or less graphitic carbon during the heat treatment. Interestingly, Choi and Kang [122] reported that dextrin might be preferable to glucose and sucrose to reduce the hygroscopicity of spray-dried powders (Figure 5).

As can be seen in Table 5, carbon nanotubes (CNT) are a possible choice amongst conducting carbons that can be added to a solution/suspension before spray-drying. Most often, CNTs are added as a (commercial) dispersion. Sometimes there is little or no information about the characteristics of the CNTs (size distribution, residues of synthesis, dispersing agents, etc.) and even where reference and provider are reported it often turns out that the corresponding commercial datasheets are less than detailed. To some extent, the same comments apply to carbon blacks, although they are usually bought in powder form and easier to characterize. Also, they can be selected amongst the relatively well-known references commonly used for electrode formulation. Since pristine graphene does not disperse in water-based solution/suspensions, graphene oxide (GO) nanosheets suspensions (about which even less is usually known than in the case of CNT) are used and reduction to graphene (reduced graphene oxide—RGO) is achieved by heat treatment or, much less often, by chemical reduction with hydrazine vapor [37,60,83].

Similar principles apply to electrode materials that are made up of carbon only, typically as negative electrodes for Li-ion or Na-ion batteries [25,26,27,28,29,30,31,33,35] or as hosting material in Li-O_2_ or Li-S batteries [23,24,32,34].

One of the electrode materials for which the broadest variety of carbon sources has been investigated is silicon, because the formation of Si/C composites is one of the most common strategies to buffer the expansions/contractions of Si during electrochemical cycling vs. Li.

Table 6 provides brief descriptions of the suspension compositions and post-spray-drying (post-SD) treatments. The last column reports the percentage of Si in the final Si/C composite materials. The references are sorted into categories depending on the role of spray-drying in the experimental procedure.

It can be seen that in many cases, the suspension formulation includes a combination of several carbons or carbon precursors. In some cases (Category C in Table 6), Si is mixed with carbon and carbon precursors in a first spray-drying step, then, the heat-treated composites are again mixed with carbon in a second spray-drying step.

## 5. Post-Processing of the Spray-Dried Precursors

Spray-drying can be used as a shaping-only method to prepare microspheres and/or as a mixing method for components that do not require further transformation. However, the spray-dried powder is often an intermediate in the synthesis procedure. The very common case of a heat treatment is considered in Section 5.1 while more complex post-spray-drying procedures are described in Section 5.2.

### 5.1. Heat Treatment

Spray-dried powders often require a heat treatment to transform into the final phase. Depending on the composition of the as-sprayed material, this heat treatment involves thermal decomposition of precursors and/or solid state diffusion and/or crystallization. Thermal analysis (TGA/TDA) and X-ray diffraction are standard characterization techniques helping to optimize the temperature and duration of the heat treatment. Regarding the inorganic active material, heat treatment usually aims at a homogeneous, single-phase composition. Occasionally (see Composites at the end of Table 1), the precursor obtained by spray-drying of a solution is deliberately meant to crystallize into a mixture of two active phases, for example LiFePO_4_-Li_3_V_2_(PO_4_)_3_ [359,360,361,362,363].

In the case of electrode compounds in which elements are not at their maximum oxidation state, the solution, suspension or spray-dried precursor may contain species susceptible to oxidation. If necessary, oxidation in solution can be suppressed by reducing additives, complexation and/or removal of dissolved oxygen by degassing. During spray-drying in air, oxygen might lead to some oxidation but most authors do not pay much attention to this effect, due to the short residence time in the spray-dryer. On the contrary, the atmosphere during the heat treatment step is a parameter of major importance to prevent oxidation or even promote reduction (typically in Ar/H_2_ with 2 to 10 vol % H_2_). This is illustrated by Categories B and C in Table 6 for the case of the synthesis of Si/C composites: oxidation of Si and existing carbon (such as CNT, carbon black, etc.) must be prevented and carbon precursors should transform into more or less graphitized carbon. An overview of the heat treatments reported in Table 6 (B&C) reveals a rather broad range of temperatures and atmospheres.

### 5.2. More Complex Post-Processing

In some cases, the spray-dried material is only an intermediate and is used as one of the reactants in an ulterior synthesis step. An unlithiated spray-dried (hydr)oxide of several transition metals can be mixed with a lithium salt to provide the electrode material by solid state reaction (see for example [214,376]). In a work by Wang et al. [377], a spray-dried composite of graphene-polyacrylonitrile was reacted with elemental sulfur in a nitrogen atmosphere at 300 °C. Similarly, Liu et al. [378] used mesoporous carbon microspheres prepared by spray-drying as a host for selenium. Oxides in spray-dried metal oxide/carbon composites can be transformed into sulfides or selenides by reaction with appropriate gaseous atmospheres (thiourea in Ar/H_2_ [114,375,379] or Se in Ar/H_2_ [367]). Wang et al. [380] reported the impregnation of molten lithium in CNT spray-dried spheres. Some authors [48,49] proposed the reduction of SiO_2_ in spray-dried SiO_2_/CNT composites by reaction with magnesium metal followed by dissolution of MgO in HCl.

The powders obtained in the spray-drying step can also be dispersed in a solution/suspension that is expected to form a coating of a different phase by sol-gel process (ZrO_2_, TiO_2_ or Al_2_O_3_ on LiNi_1/3_Co_1/3_Mn_1/3_O_2_ [150,156]; Li_4_Ti_5_O_12_ on LiMn_2_O_4_ [190,309] or LiFePO_4_ [298]), by evaporation of the solvent (LiFePO_4_ on Li_3_V_2_(PO_4_)_3_ [336], LiMnPO_4_ [168] or CeO_2_ [178] on Li_1.17_Ni_0.25_Mn_0.58_O_2_), or by another spray-drying step (LiCoO_2_ on LiMn_2_O_4_ [381]; Li_3_PO_4_ on Li_4_Ti_5_O_12_ [219]; LiF on Si [72]).

Chemical vapor deposition (CVD) is sometimes used to create an additional carbon layer [49,64,71,95,352] or to grow carbon nanotubes/nanofibers if the necessary catalyst was included in the spray-drying step [76,118]. In a work by Shi et al. [382], sacrificial spray-dried layered double oxide (LDO) microspheres act as a template and a catalyst for the CVD growth of graphene; chemical etching of LDO yields a 3D graphene host for sulfur in Li-S batteries. Zhang et al. [383] reported CVD growth of a Si/C layer on graphitized spray-dried carbon black porous microspheres.

The variety of post-spray-drying processing can be further illustrated by the examples in Category D of Table 6, focusing on spray-dried Si.

## 6. Microstructure

This section is devoted to the microstructural aspects of spray-dried materials. As already mentioned in the introduction, these aspects are extremely important in the case of electrode materials. Basically, anything that favors (i) the penetration of the liquid electrolyte in the electrode material; (ii) short solid state diffusion paths of Li^+^/Na^+^ ions or (iii) fast transport of electrons is expected to improve the cycling performance. However, it should be kept in mind that high porosity or high content of compounds that do not store charge (e.g., carbon added to facilitate electron transport) will be paid for in terms of energy density (per volume or per mass, respectively).

Here the discussion focuses on the morphology of the individual granules (as-sprayed or after heat treatment) and on possibilities to influence it by various deliberate strategies. It is well-known that spray-drying tends to produce microspheres (Figure 2a) as the result of droplet drying. However, fast drying can also result in the precipitation/solidification of thin crusts leading to hollow or collapsed spheres (Figure 2b,c), depending on the mechanical strength of the crust. Hydrodynamic and/or visco-elastic effects are believed to be at the origin of more exotic shapes such as the “doughnut” particles [384]. The reader is referred to the review by Nandiyanto and Okuyama [9] for a catalogue and discussion of possible morphologies.

**Figure 6:**
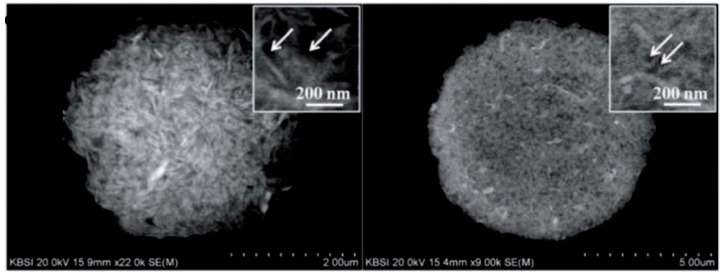
SEM images of cross-sections in (**left**) Co_3_O_4_ and (**right**) CoO–carbon composite powders. Both were obtained by a sequence of solution spray-drying—heat treatment in N_2_—milling—suspension spray-drying—heat treatment (in air for Co_3_O_4_, in N_2_ for CoO/C). (Adapted from [100] by permission of The Royal Society of Chemistry).

The concentration/solid loading of the solution/suspension (see for example [236]) and the spray-drying experimental parameters (equipment, inlet/outlet temperature, atomization parameters) all influence the average size, size distribution, and shape of spray-dried granules. Spray-drying of a solution often yields hollow, thin-shell spheres; the inside volume can be considered as lost space from the point of view of energy density. Breaking these spheres by grinding/milling and shaping the broken pieces into denser—but still porous—spheres by spray-drying of a suspension allows for a large gain in volumic efficiency (see Figure 6 adapted from [100]).

**Figure 7:**
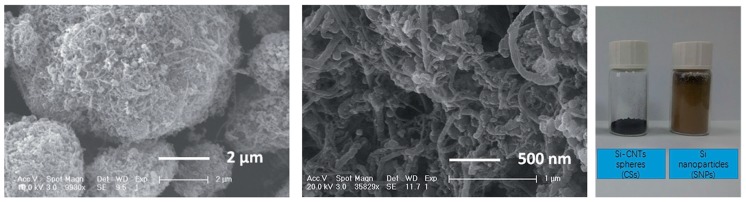
(**left** and **middle**) SEM images of Si/carbon nanotubes (CNT) composite microspheres; (**right**) Comparison of the volume occupied by equivalent masses of Si/CNT spray-dried composite spheres and of original Si nanoparticles. (Adapted from [55]—Published by The Royal Society of Chemistry under CC BY 3.0—https://creativecommons.org/licenses/by/3.0/).

Spray-drying of suspensions is indeed recognized as a technique favoring packing efficiency, as illustrated in Figure 7 (adapted from [55]), showing a comparison of the volume occupied by equivalent masses of Si/CNT spray-dried composite spheres and of original Si nanoparticles.

**Figure 8:**
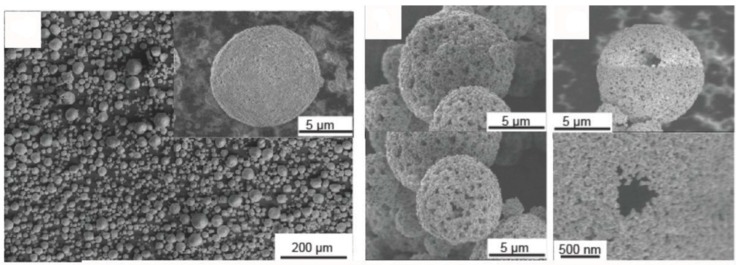
Li_4_Ti_5_O_12_ spray-dried granules after heat treatment in air to decompose the organic templates: (**left**) nanoporous microspheres obtained from spray-drying with 3 wt % cellulose; (**middle**) macroporous spheres obtained from spray-drying with polystyrene beads as template and (**right**) microspheres with channel structures obtained from spray-drying with carbon fiber templates. (Reproduced from [234] under CC BY 4.0—https://creativecommons.org/licenses/by/4.0/).

The microstructure and porosity of as-sprayed granules can further evolve during heat treatment due to decomposition/graphitization of organics, crystallization, crystal growth or sintering. The porosity created by the decomposition of organics during a heat treatment in air is expected to help penetration of the electrolyte in the electrode material. Some authors have proposed a hard templating strategy based on polystyrene beads [234,260,318] to introduce controlled macroporosity. For example Nowack et al. [234] investigated the combined effects of nanoporosity (created by thermal decomposition of cellulose) and macroporosity (created by thermal decomposition of polystyrene spheres or carbon fibers) in Li_4_Ti_5_O_12_ spray-dried granules (Figure 8 reproduced from [234]).

Similar strategies rely on other sacrificial phases, such as SiO_2_ spheres [32,34,385], in situ formed metal [128] or NaCl [46,74,80,82] particles, all of which are removed at a later stage by chemical etching (SiO_2_, metals) or washing (NaCl).

**Figure 9:**
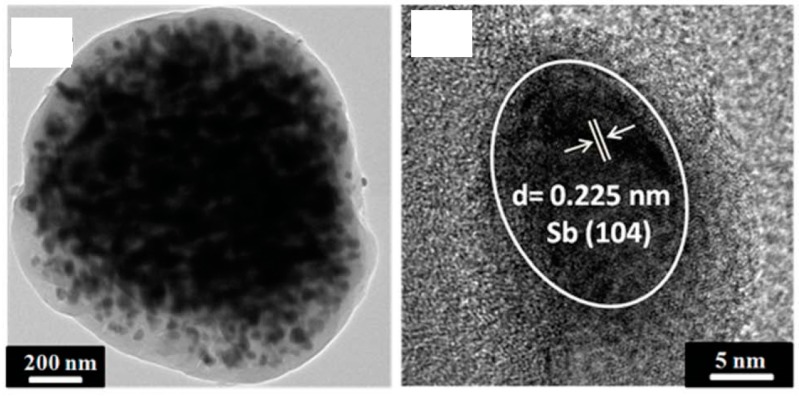
Sb nanoparticles embedded in carbon matrix: (**left**) transmission electron microsopy (TEM) image; (**right**) high resolution TEM (HRTEM) image. (Adapted from [40] with permission from The Royal Society of Chemistry).

As already explained in Section 4 and Section 5, spray-dried electrode materials are frequently designed as composites with carbon in order to improve electron transport and/or buffer volume variations. Figure 9 shows an example of Sb nanoparticles embedded in a carbon matrix formed by carbonization of the organic precursor during heat treatment of the spray-dried precursor in inert atmosphere.

**Figure 10:**
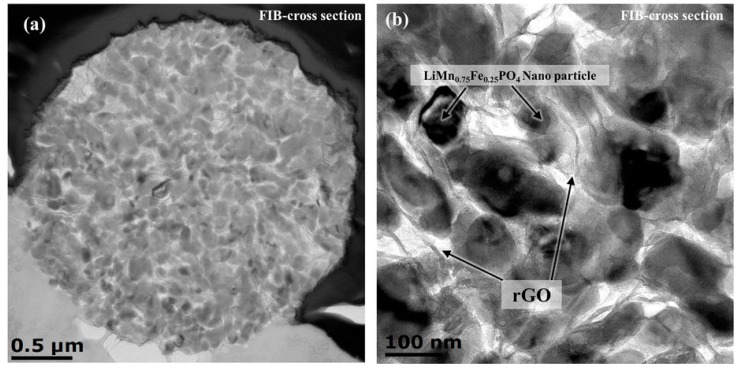
(**a**,**b**) Cross-sectional TEM images of LiMn_0.75_Fe_0.25_PO_4_/reduced graphene oxide composite microsphere. (Adapted from [310] under CC BY 4.0—https://creativecommons.org/licenses/by/4.0/).

**Figure 11:**
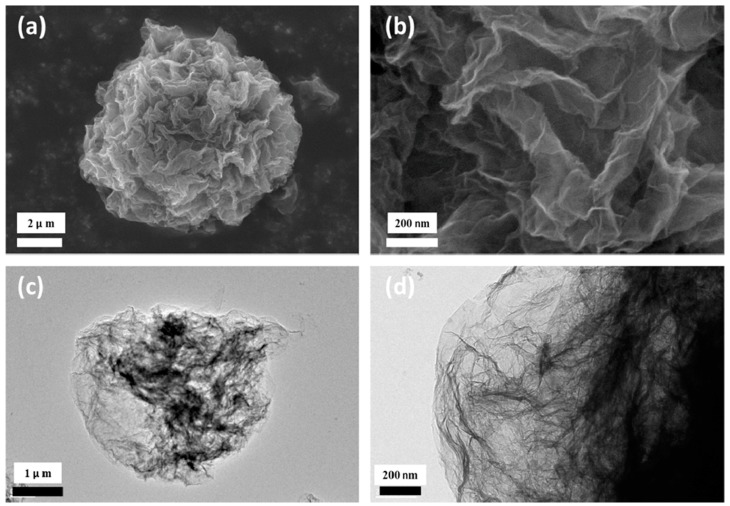
Graphene network after chemical etching of the Na_3_V_2_(PO_4_)_3_ phase: (**a**,**b**) SEM images; (**c**,**d**) TEM images. (Reproduced with permission from [344]. Copyright (2017) American Chemical Society.).

When carbon is added as CNT, carbon black, graphite or graphene oxide in the solution/suspension before spray-drying, there is an (often implicit) assumption that the distribution of carbon in the granules will be of sufficient homogeneity. In the case of composites with reduced graphene oxide, some authors have been able to supplement the usual SEM and TEM images (see Figure 7 for a CNT example) by cross-sectional TEM (Figure 10—adapted from [310]) or imaging of the graphene network after chemical etching of the inorganic phase (Figure 11—adapted from [344]).

**Figure 12:**
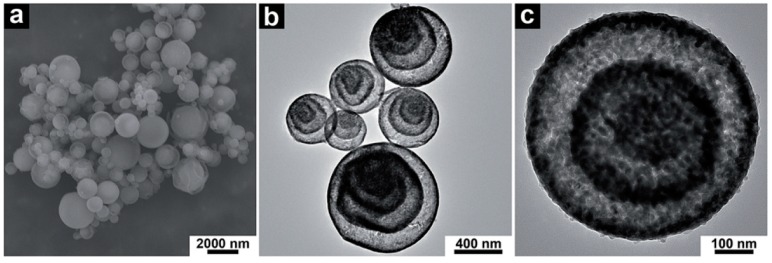
Hematite Fe_2_O_3_ multi-shelled hollow spheres obtained by heat treatment of precursors spray-dried from an iron(III) citrate and sucrose solution: (**a**) SEM image; (**b**,**c**) TEM images. (Adapted from [107] with permission of The Royal Society of Chemistry).

This overview of morphologies cannot be exhaustive. The examples shown in Figure 6, Figure 7, Figure 8, Figure 9, Figure 10 and Figure 11 correspond to morphologies that retain a (roughly) spherical appearance, but Figure 2b,c should remind the reader that crumpled morphologies are also common. As a final illustration of the microstructural variety, Figure 12 displays a more unexpected, multi-shelled morphology which has been reported and studied by several groups [101,107,139]. Yolk-shell granules [103,122,136] are a less extreme case of a similar phenomenon.

## 7. Electrochemical Properties

The overwhelming majority of spray-dried materials reported in the literature for Li-ion and Na-ion batteries are used as electrode materials. Amongst the few exceptions are (i) Li_1.3_Al_0.3_Ti_1.7_(PO_4_)_3_ [338] which is used as a solid state electrolyte and (ii) La_2_O_3_ [113] or CeO_2_ [98] hollow spheres which are coated on the separator of Li-sulfur batteries and are supposed to block lithium polysulfides and act as a catalyst for the sulfur redox reaction.

Literature on spray-dried materials for positive or negative electrodes follows the general trend: the largest number of publications concerns materials for Li-ion batteries but research on compounds for Na-ion batteries is increasing strongly in recent years. Regarding emerging technologies, spray-drying is receiving interest as a tool to prepare porous carbon hosts for sulfur/selenium in Li-sulfur [23,24,34,37,38,39,116,120,128,377,385] or Li-selenium [83,378] batteries. Similarly, reduced graphene oxide microspheres with high surface area were tested in Li-air batteries [32]. In the field of “beyond Li/Na” technologies, Na_3_V_2_(PO_4_)_3_/C [343] and Li_3_VO_4_/C [261] obtained by spray-drying have recently been mentioned in research on Mg-ion batteries.

As explained at the end of the introduction, the main focus of this review is on guidelines for the formulation of spray-drying feedstock solutions/suspensions and how it can affect microstructure. In the following of this section, a few examples are selected to illustrate the link between formulation, microstructure and electrochemical properties. As a complement, Table A3 in Appendix A lists values of experimental discharge capacities after 50 cycles.

**Figure 13:**
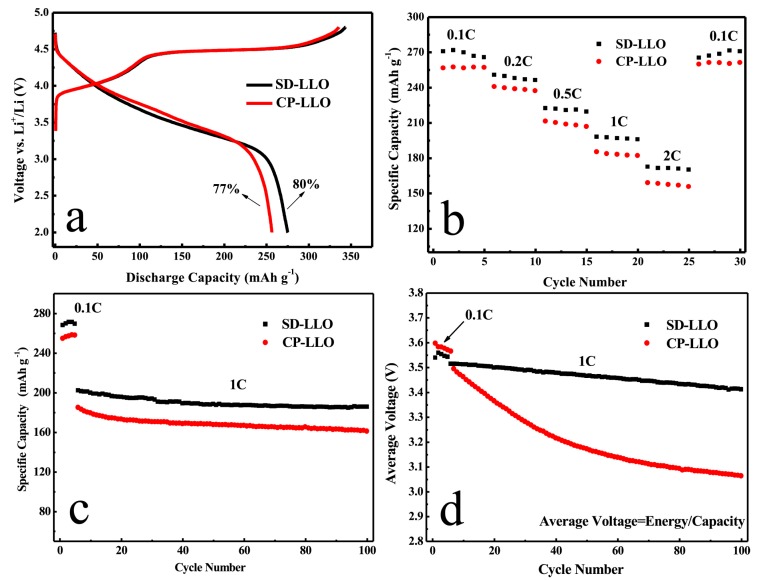
Comparison of two samples of Li-rich oxide 0.5Li_2_MnO_3_-0.5LiMn_1/3_Ni_1/3_Co_1/3_O_2_ obtained by a spray-drying procedure (SD-LLO) or by a dry mixing procedure (CP-LLO)—see text for details. (**a**) First cycle charge/discharge profiles; (**b**) Rate performance; (**c**) Cycling performance between 2 and 4.8 V; (**d**) Average discharge voltage as a function of cycle number during cycling. (Reproduced from [149]. Copyright (2015), with permission from Elsevier).

The first examples concern layered oxides, including Li-rich compositions sometimes written as xLi_2_MnO_3_-(1−x)LiMO_2_ (M = Ni, Co, Mn, …), which are studied because of their high theoretical reversible capacity (above 250 mAh/g). Hou et al. [149] reported the synthesis of 0.5Li_2_MnO_3_-0.5LiMn_1/3_Ni_1/3_Co_1/3_O_2_ (=Li_1.2_Mn_0.54_Ni_0.13_Co_0.13_O_2_) by heat treatment of a precursor obtained by spray-drying of an aqueous suspension of Li_2_CO_3_ and a coprecipitated metal hydroxide (SD-LLO sample). For comparison, another sample was prepared by heat treatment of a dry mixture of Li_2_CO_3_ and coprecipitated metal hydroxide (CP-LLO sample). The authors found that the spray-drying procedure was more efficient to promote the homogeneity of the distribution of metal cations in the final oxide and resulted in better electrochemical performance (see Figure 13 reproduced from [149]). In particular, the decrease in average cell voltage was much less marked (Figure 13d), which was considered as an indication of the better stability of the layered structure against transformation into spinel structure on cycling [149].

The work by Hou et al. [149] described above can be considered as a demonstration of the superiority of wet mixing over dry mixing. In a study of Chen et al. on LiNi_0.8_Co_0.15_Al_0.05_O_2_ [143], a suspension of a ball-milled precursor was dried either by spray-drying (SD-NCA sample) or by common drying (CD-NCA sample). The mixing by ball milling is the same in the two samples so that the much better electrode performance of the SD-NCA sample (e.g., a capacity retention of 75% after 500 cycles at 2 C, against only 12% for the CD-NCA sample) can be attributed to a more favorable microstructure induced by spray-drying.

These two examples highlight positive features of the spray-drying of suspensions. This should not mask the fact that spray-drying of suspensions is a variant of solid state synthesis and is, therefore, subject to the usual limitations associated to diffusion lengths in the solid state. This was recently illustrated in a work by Wang et al. [189] where the formation of Li[Li_0.2_Mn_0.54_Ni_0.13_Co_0.13_]O_2_ was followed by in-situ high-energy X-ray diffraction during the heat treatment. Irregularities in the temperature dependence of the crystallographic cell parameters and the presence of secondary phases were observed in the case of a precursor obtained by spray-drying a ball-milled suspension of the individual oxides and carbonates (Li_2_CO_3_, MnCO_3_, Co_3_O_4_ and NiO). As could be expected, these irregularities and the content in secondary phases decreased when the suspension was prepared by ball-milling a precalcined mixture. Minimizing diffusion lengths is the usual reason to turn from solid state synthesis to solution routes. In the case of spray-drying, this means going from suspensions to solutions. For example, Watanabe et al. [174] could obtain a discharge specific capacity of 275 mAh/g for Li_1.2_Mn_0.58_Ni_0.18_Co_0.03_O_2_ obtained by spray-drying of a solution of acetates in aqueous citric acid.

In the case of compounds with relatively low intrinsic electronic conductivity, the microspheres obtained by spray-drying are often too large for good performance. One of the works demonstrating this effect was published by Nakahara et al. [233] in 2003, where the authors compare as-obtained (LT-2 sample) and ball-milled (LT-FP sample) Li_4_Ti_5_O_12_ prepared by spray-drying and heat treatment of an aqueous suspension of LiOH and TiO_2_. The 5–10 µm sintered granules were broken by ball-milling into sub-micron particles; electrodes were prepared by mixing with acetylene black and PVDF and tested in half-cells against lithium metal. The rate capability test showed that the discharge capacity of the ball-milled LT-FP sample decreased by less than 15% when going from 0.15 C to 10 C, whereas the discharge capacity of the LT-2 sample had already decreased by more than 40% at 5C.

As already mentioned in the previous sections, another way to deal with the issue of electronic conductivity is to form/include conductive carbon in the spray-dried material. This strategy is relevant whenever the subsequent heat treatment can be carried out in non-oxidizing atmosphere. For example, soluble precursors of carbon are commonly added to suspensions for the preparation of LiFePO_4_/C composites. In a work by Liu et al. [283], LiFePO_4_ with 2.5 wt % C was obtained by heat treatment in N_2_ of a precursor prepared from an aqueous suspension of Li_2_CO_3_ and FePO_4_ into which glucose had been dissolved. The authors compared spray-drying with microwave drying through testing of 14500-type cylindrical batteries with a graphite negative electrode and attributed the ~10% better performance of the spray-dried material to the higher compaction density of the electrode (2.55 g/cm³) that could be reached thanks to the favorable microstructure.

**Figure 14:**
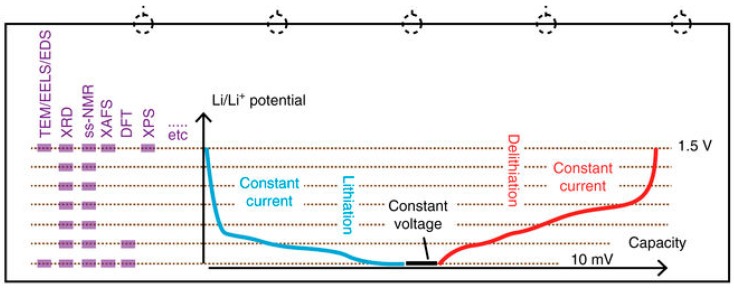
Overview of a structural characterization study conducted on spray-dried Si/C composites at different stages during individual cycles. The set of characterizations was repeated every 20 cycles. (Reproduced from reference [79] under CC BY 4.0—https://creativecommons.org/licenses/by/4.0/).

In the previous example, the LiFePO_4_ active material was formed during the heat treatment. In other cases, spray-drying is used to create a composite of carbon with an existing active material, such as silicon. As seen in Table 6, there is an impressive variety of carbon sources to choose from, but comparison is difficult because of the wide range of Si/C ratio in the final materials. In view of guiding the development of Si/C negative electrodes with high Si content, Ogata et al. [79] used two spray-dried Si/C composites (Si/flake graphite/CNT with 54 wt % Si and Si/flake graphite with 87 wt % Si—both are extensively characterized in the Methods section of ref. [79]) as the reference materials for a very detailed study of the phenomena governing coulombic efficiency. This was done by cycling the materials at different depth of discharge in order to probe the volume change of the amorphous phase and/or the amorphous-crystalline transformations. As shown in Figure 14 (reproduced from [79]), a broad range of techniques were used to characterize the (micro)structure and composition at different stages of individual cycles.

**Figure 15:**
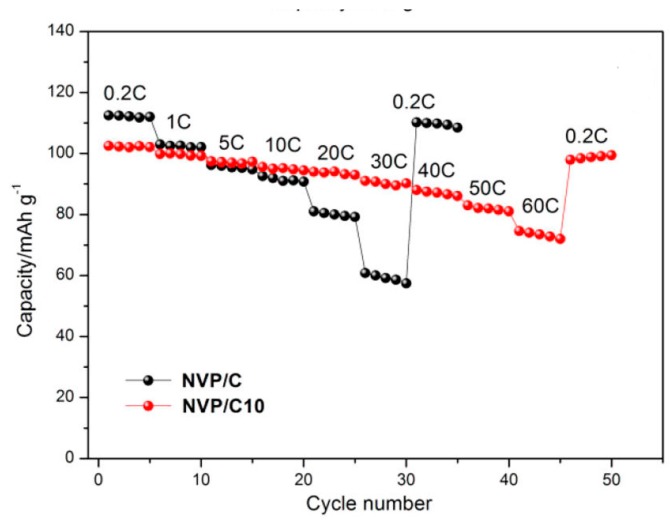
Rate capability of Na_3_V_2_(PO_4_)_3_ with 10 wt % CNT (NVP/C10) and without CNT (NVP/C). The electrodes were cycled vs. Na in the 2.0–3.8 V voltage range. Both samples were obtained by heat treatment of a spray-dried precursor prepared from a citric acid solution of NaHCO_3,_ NH_4_VO_3_ and NH_4_H_2_PO_4_ into which CNT were dispersed in the case of the NVP/C10 sample. (Reproduced with permission from [342]. Copyright (2018) American Chemical Society).

From a chemical point of view, the most complex case is probably when a solid form of carbon is dispersed in a solution of several inorganic salts. This is typically the case for the spray-drying synthesis of phosphates or fluorophosphates from solutions where carbon nanotubes or graphene oxide are added to provide electronic conductivity. In our work on Na_3_V_2_(PO_4_)_2_F_3_/CNT [93], we found that an excess amount of NaF was necessary to prevent the formation of a small amount of fluorine-free Na_3_V_2_(PO_4_)_3_ secondary phase, suggesting that the addition of CNT to the solution interferes a little with the inorganic components. Conversely, the high concentration of several ions in the solution is supposed to affect the dispersion of carbons, although this effect has not yet been studied in such very complex situations. This might be one of the reasons why we observed an inhomogeneous distribution of carbon black (CB) in spray-dried granules of Na_2_FePO_4_F/CB [89], leading to a drop of 60% in discharge capacity compared to Na_2_FePO_4_F/CNT composites with similar carbon content [87,89]. A work by another group [342] on the fluorine-free alluaudite phosphate Na_3_V_2_(PO_4_)_3_ (with the drawback of a lower operating voltage) confirms that excellent rate capability is possible for a Na_3_V_2_(PO_4_)_3_/CNT composite (Figure 15, reproduced from [342]). Along the same lines, Table 5 shows that graphene oxide (reduced during post-treatment) is becoming a popular choice for many phosphates, as exemplified by the results for NaTi_2_(PO_4_)_3_/RGO [340], where the discharge capacity decreases by less than 10% when going from 0.1 C to 30 C rate (130 mAh/g at 0.1 C, 118 mAh/g at 30 C).

## 8. Concluding Remarks

It should be clear from the preceding sections that the term “spray-drying” covers many different realities. Reasons for using spray-drying are varied since it can be used as a tool for mixing, shaping, or synthesizing (or combining several of these objectives simultaneously).

In many cases, spray-drying is not really a rival to other routes but rather a way to bring a laboratory-scale procedure to the next level in terms of production quantities, reproducibility, and control of agglomeration. This is true, for example, for many solid state reaction syntheses on the condition that the starting materials are not soluble in the liquid medium of the suspension. This can also be the case for sol(ution)-gel routes, taking into account that the increase in drying speed might modify some characteristics by comparison with a conventionally-dried gel. More generally, spray-drying can be considered in all cases where no problem comes from the fact that, except for volatiles, everything that is injected in the spray-dryer turns up in the as-spray-dried powder.

In other cases, spray-drying offers new opportunities, such as the dispersion of carbon in active material or the possibility offered by the droplet scale to use a simple solvent evaporation route (which, in other conditions, would result in unacceptably large composition inhomogeneities).

Spray-drying is commonly used in industry in many fields of applications. The 300+ publications referenced in this review demonstrate that the potential of spray-drying is increasingly recognized in the academic community for the synthesis of electrode materials from lab- to pilot-scale quantities.

However, the apparent simplicity of the spray-drying concept should not mask the fact that choices regarding the formulation of solutions/suspensions and the selection of experimental spray-drying parameters decisively affect the characteristics of the final material. Optimization of the parameters of the subsequent heat treatment is also very important but cannot alter drastically the microstructural properties. It is the hope of the authors that this review can contribute to a realization that making the most of spray-drying requires a considered choice amongst possible strategies and careful consideration of the solution/suspension formulation.

## Figures and Tables

**Table 1:** Bibliographical overview.

Compound Types, Formulas and References
**Borates** LiMnBO_3_ [20], LiFeBO_3_ [21], Li(Fe,Ni)BO_3_ [22]
**Elements** C [23,24,25,26,27,28,29,30,31,32,33,34,35], P [36], S [37,38,39], Sb [40], Si [41,42,43,44,45,46,47,48,49,50,51,52,53,54,55,56,57,58,59,60,61,62,63,64,65,66,67,68,69,70,71,72,73,74,75,76,77,78,79,80,81], Sn [82], Se [83]
**Fluorides** Li_2_TiF_6_ [84], Li_2_NiF_4_ [85], Li_3_FeF_6_ [86]
**Fluorophosphates** Na_2_FePO_4_F [87,88,89], Na_2_MnPO_4_F [90,91], Na_2_CoPO_4_F [92], Na_3_V_2_(PO_4_)_2_F_3_ [93], Na_3_V_2_O_2_(PO_4_)_2_F [94]
**Organic salts** Dilithium terephtalate Li_2_C_8_H_4_O_4_ [95], Disodium terephtalate Na_2_C_8_H_4_O_4_ [96], Disodium 2,5-dihydroxy-1,4-benzoquinone Na_2_C_6_H_2_O_4_ [97],
**Oxides M_x_O_y_** CeO_2_ [98], CoO_x_ [99], CoO [100], Co_3_O_4_ [100,101,102], Cr_2_O_3_ [103], CuO [104,105,106], Fe_2_O_3_ [107,108,109,110], GeO_x_ [111], GeO_2_ [112], La_2_O_3_ [113], MnO [114], MoO_3_ [115], Nb_2_O_5_ [116], NiO [117], SiO [118,119], SiO_2_ [120,121], SnO_2_ [122,123,124], TiO_2_ [125,126,127,128,129,130,131,132,133], V_2_O_5_ [134]
**Oxides M_x_M’_y_O_z_** ZnFe_2_O_4_ [135,136], Mn_0.5_Co_0.5_Fe_2_O_4_ [137], NiCo_2_O_4_ [138], (Ni,Co)O_x_ [139], Cu_1.5_Mn_1.5_O_4_ [140], NiMoO_4_ [141], TiNb_2_O_7_ [142]
**Oxides Li_x_M_y_O_z_** Layered oxides Li_x_M_y_O_2_ (M = Li, Ni, Co, Mn, Al, …) [143,144,145,146,147,148,149,150,151,152,153,154,155,156,157,158,159,160,161,162,163,164,165,166,167,168,169,170,171,172,173,174,175,176,177,178,179,180,181,182,183,184,185,186,187,188,189]—see Table 3 for compositions LiMn_2_O_4_ [190,191,192,193,194,195,196,197,198,199], Co-doped LiMn_2_O_4_ [200,201], Cr-doped LiMn_2_O_4_ [202,203], Ni-doped LiMn_2_O_4_ [204,205] LiNi_0.5_Mn_1.5_O_4_ [206,207,208,209,210], Ti-doped LiNi_0.5_Mn_1.5_O_4_ [211], Fe,Ti-doped LiNi_0.5_Mn_1.5_O_4_ [212], Ru,Ti-doped LiNi_0.5_Mn_1.5_O_4_ [212], Co-doped LiNi_0.5_Mn_1.5_O_4_ [213,214,215] Li_4_Ti_5_O_12_ [216,217,218,219,220,221,222,223,224,225,226,227,228,229,230,231,232,233,234,235,236,237,238,239,240,241,242,243,244,245,246,247,248,249], Li_3.98_Al_0.06_Ti_4.96_O_12_ [250], Li_4+x_Ti_4.95-x_Nb_0.05_O_12-d_ [251] Li_x_V_3_O_8_ [252,253,254,255,256,257], Li_3_VO_4_ [258,259,260,261], Li_4_Mn_5_O_12_ [262]
**Oxides Na_x_M_y_O_z_** Na_2/3_Ni_1/3_Mn_2/3_O_2_ [263,264], Na_2_Ti_3_O_7_ [265], Na_4_Mn_9_O_18_ [266,267]
**Phosphates** FePO_4_ [268,269,270] LiFePO_4_ [271,272,273,274,275,276,277,278,279,280,281,282,283,284,285,286,287,288,289,290,291,292,293,294,295,296,297,298,299,300,301,302,303,304,305,306,307,308,309], Li(Fe,Mn)PO_4_ [310,311,312,313,314,315,316,317,318,319,320], LiMnPO_4_ [321,322], Li(Mn_0.85_Fe_0.15_)_0.92_Ti_0.08_PO_4_ [323] LiVOPO_4_ [324] Li_3_V_2_(PO_4_)_3_ [325,326,327,328,329,330,331,332,333,334,335,336], Li_3_(V,Al/Fe)_2_(PO_4_)_3_ [337], electrolyte Li_1.3_Al_0.3_Ti_1.7_(PO_4_)_3_ [338] NaTi_2_(PO_4_)_3_ [339,340], Na_3_V_2_(PO_4_)_3_ [341,342,343,344,345], Na_3_V_2-x_Cu_x_(PO_4_)_3_ [346],
**Pyrophosphates** Na_2_FeP_2_O_7_ [347], SnP_2_O_7_ [348]
**Silicates** Li_2_FeSiO_4_ [349,350], Li_1.95_Na_0.05_FeSiO_4_ [351], Li_2_Fe_0.5_V_0.5_SiO_4_ [352]
**Sulfides and selenides** MnS [114], MoS_2_[353], FeSe_2_ [354]
**Composites (not with carbon)** Sn–Sn_2_Co_3_@CoSnO_3_–Co_3_O_4_ [355], Fe_2_O_3_-SnO_2_ [356], LiNi_0.5_Mn_1.5_O_4_-Li_7_La_3_Zr_2_O_12_ [357], 3Li_4_Ti_5_O_12_.NiO [358], LiFePO_4_-Li_3_V_2_(PO_4_)_3_ [359,360,361,362,363], LiMnPO_4_-Li_3_V_2_(PO_4_)_3_ [364,365], Si-FeSi_2_-Cu_3.17_Si [366], MoS_2_–Ni_9_S_8_ [367], MoSe_2_–NiSe(–NiSe_2_) [367]

**Table 2:** Spray-drying synthesis of active materials involving organic or partially organic suspensions.

Liquid	Active Material
Ethanol	S [38], Si [42,45,47,52,57,58,69,76], SiO [118], SiO_x_ [371], TiO_2_ [129,130], Li_x_Mn_1/3_Co_1/3_Ni_1/3_O_2_ [150], Li_4_Ti_5_O_12_ [218], LiFePO_4_ [273,277,289], Li_3_V_2_(PO_4_)_3_ [326]_,_ LiFePO_4_-Li_3_V_2_(PO_4_)_3_ [359], Li_2_Fe_0.5_V_0.5_SiO_4_ [352]
Alcohol (unspecified)	Li_4_Ti_5_O_12_ [231,232]
Ethanol-water	C [23], Si [54,60,65,72], SiO_2_ [120], SnO_2_ [123], TiO_2_ [132], LiMn_2_O_4_ [199], Li_4_Ti_5_O_12_ [229,238,241,243,250,251], Na_2_Ti_3_O_7_ [265], LiFePO_4_ [292]
Alcohol-water	Si [73,78]
Other liquid(s)	DMF for Sb/C [40], EG for Si/C [43], Ethylene glycol—cyclohexane for ZnFe_2_O_4_ [135], THF for Si/C [44,58], water-THF for Li_3_PO_4_-coated Li_4_Ti_5_O_12_ [219]

**Table 3:** Spray-drying for layered oxides AMO_2_ (A = Li^+^, Na^+^; M = one/several of Li, Ni, Mn, Co, Al, …).

	Li	Co	Ni	Mn	other	Comments
**SPRAY-DRYING OF SOLUTIONS**						
**A. Spray-drying of aqueous solution of nitrates and/or acetates**						
Duvigneaud et al. [145]	1	0.18 − y	0.82	-	Al	+ polyvinyl alcohol
He et al. [146]	1	0.105	0.35	0.545	Cr	0 to 6% Cr
He et al. [148]	✓	✓	✓	✓	-	-
Kim et al. [151]	1 + x	1/3	1/3	1/3	-	-
Kim et al. [152]	1 + x	1 − 2z	z	z	-	x = 0–0.1; z = 0.1–0.4
Kim et al. [187]	1 + x	0.4	0.3	0.3	-	-
Konstantinov et al. [153]	1	1	-	-	-	-
Li et al. [154,156]	1	1/3	1/3	1/3	-	-
Li et al. [157]	1	1/3	1/3	1/3	-	+ LiF
Li et al. [160]	1	1	-	-	-	+ polyethylene glycol
Liu et al. [166]	1	1/3	1/3	1/3	-	+ PVA
Wang et al. [263]	Na_2/3_	-	1/3	2/3	-	-
Wang et al. [172]	1.57	1/6	1/6	2/3	-	-
Wang et al. [173]	1 + x	1 − x	-	x	-	-
Wu et al. [175]	1	0.2	0.8	-	-	-
Yue et al. [179,180]	1 + x	0.2	0.6	0.2	-	x = 0; 0.04
Zhang et al. [183]	1 + x	-	0.5 − x/2	0.5 − x/2		x = 0–0.2
Zhang et al. [186]	1	1/3	1/3	1/3	-	-
Zhao et al. [264]	Na_2/3_	-	1/3	2/3	-	Followed by Li^+^/Na^+^ ion exchange
**B. Spray-drying of aqueous solution of salts dissolved in aqueous citric acid**						
Li et al. [158]	✓	-	✓	✓	Fe	nitrates
Sun et al. [171]	✓	✓	✓	✓	-	acetates
Watanabe et al. [174]	1.2	0.03	0.18	0.58	-	acetates
Zhang et al. [184,185]	✓	-	✓	-	Ti	LiOH, Ni acetate and [NH_4_]_2_[Ti(C_2_O_4_)_3_]
**C. Spray-drying of aqueous solution of citrates**						
Li et al. [155]	1	2x	0.5 − x	0.5 − x	-	x = 0–0.1
Qiao et al. [169]	1.17	-	0.25	0.58 − x	Sn	x = 0–0.05
Yuan et al. [178]	1.17	0.05	0.2	0.58	-	-
**D. Spray-drying of aqueous solution (others)**						
Li et al. [159]	1	1	-	-	-	hydroxides dissolved in polyacrylic acid solution
Oh et al. [167]	1	0.2	0.8	-	-	hydroxides and carbonate dissolved in acrylic acid solution
**SPRAY-DRYING OF SUSPENSIONS**						
**E. Spray-drying of an aqueous suspension to mix reactants**						
Hou et al. [149]	1.2	0.13	0.13	0.54	-	Li_2_CO_3_ and hydroxide co-precipitate
Lin et al. [164]	1.2	-	0.2	0.6	-	carbonates and oxides
Liu et al. [165]	1	1/3	1/3	1/3	-	in situ polymerized Li polyacrylate and hydroxide co-precipitate
Wang et al. [189]	1.2	0.13	0.13	0.54		carbonates and oxides
Yue et al. [181]	1	0.2	0.6	0.2	-	Li_2_CO_3_ and hydroxide co-precipitate
**F. Spray-drying of an ethanol suspension to mix reactants**						
Hu et al. [150]	1	1/3	1/3	1/3	-	LiOH and hydroxide co-precipitate
Lin et al. [161,162]	1	1/3	1/3	1/3 − x	Zr	x = 0–0.02-carbonates and oxides
**G. Mixing of AMO_2_ active material with conductive carbon or conductive carbon precursor**						
Cheng et al. [144]	1.2	0.13	0.13	0.54	-	graphene oxide
Xia et al. [176]	1	1	-	-	-	P3DT (in CH_2_Cl_2_)
Yang et al. [177]	1.2	0.13	0.13	0.54	-	CNT
Yue et al. [182]	1	0.2	0.6	0.2	-	graphene oxide
**H. Shaping of AMO_2_ as spheres**						
Chen et al. [143]	1	0.15	0.8	-	Al	0.05% Al-starch binder

**Table 4:** Organic (macro)molecules used for the formulation of solutions/suspensions in view of spray-drying preparation of electrode materials.

Organic Compound Types, Compound and References
**Carboxylic Acids** Acetic acid [87,88,89,211,212,229,238,265], Acrylic acid [165,167], Citric acid [21,22,43,44,52,58,76,78,81,87,88,89,90,91,92,98,100,101,102,105,106,113,117,118,139,140,155,158,168,169,171,174,178,184,185,207,213,214,235,241,278,284,286,295,296,301,302,310,311,325,327,329,331,332,334,337,342,345,346,349,351,360,362,364,365], Ascorbic acid [93], Formic acid [268], Lactic acid [235], Malic acid [235], Malonic acid [235], Oxalic acid [135,227,243,248,251,278,293,311,324,335,344,365], Tartaric acid [300,303]
**Saccharides** *Monosaccharides:* Glucose [53,56,71,77,258,259,260,272,274,275,277,283,285,287,288,289,298,299,306,308,312,314,318,319,335,359,364] *Disaccharides:* Sucrose [33,46,63,64,75,80,107,108,110,120,242,279,280,281,286,293,294,296,297,302,315,316,317,320,323,326,336,343,347,348,363], Sugar [217,247] *Polysaccharides:* Cellulose [234], Starch [143,202,203,276,288,290,291,292,313], Dextrin [114,115,122,136,141,354,367], Cyclodextrin [142,299], Maltodextrin [128]
**Synthetic Polymers** Melamine-formaldehyde resin [210] Phenol-formaldehyde resin [31,42,45,47,65,273,292,371] Polyacrylic acid [159,227] Polyacrylonitrile [40] Poly(3-decaylthiophene) [176] (for thermal protection via shut-down action at 110 °C) Polyethylene glycol [160,230,280,286,299,302,304,315,316,326,359] Polystyrene-acrylonitrile [25,43] Polyvinylalcohol [59,62,67,79,121,145,166,227,269,270,274,275,286,296,307,330] Polyvinylbutyral [161,162,163,231,232] Polyvinylpyrrolidone [58,71,74,76,80,103,118,124,208,230,349,351] Triblock copolymer PEO-PPO-PEO F127 [82]
**Others** C_2_H_4_N_4_ [241], Acetylacetone [229,238], Chitosan [23,61], Diethylene glycol [237], EDTA [82], Ethylene glycol [101,139], Formamide [188], Pitch [43,44,58,73,328], Urea [269]

**Table 5:** Spray-drying synthesis of active material/carbon composites: references to publications where solid conducting carbon or graphene oxide is added to the spray-drying solution/suspension.

Carbon	Active Material
CNT	C [24], S [38], Si [48,49,55,69,73,77,79,80], SiO_x_ [371], Na_2_FePO_4_F [87,89], Na_3_V_2_(PO_4_)_2_F_3_ [93], Disodium terephtalate Na_2_C_8_H_4_O_4_ [96], Disodium 2,5-dihydroxy-1,4-benzoquinone Na_2_C_6_H_2_O_4_ [97], GeO_x_ [111]_,_ V_2_O_5_ [134], Li_x_M_y_O_2_ (M = Ni, Co, Mn, Al, …) [177], Li_4_Ti_5_O_12_ [218,248], Li_3_VO_4_ [258], Na_4_Mn_9_O_18_ [266], LiFePO_4_ [289,305], Li(Mn,Fe)PO_4_ [313,316], NaTi_2_(PO_4_)_3_ [339], Na_3_V_2_(PO_4_)_3_ [342], Li_3_V_2_(PO_4_)_3_ [329]_,_ Li_2_FeSiO_4_ [350]
Graphene oxide GO (reduced to RGO)	C [28,30,31,32], P [36], S [37,39], Se [83], Si [50,51,54,60,63,66,67,80], Na_3_V_2_O_2_(PO_4_)_2_F [94], Cr_2_O_3_ [103], CuO [105], Fe_2_O_3_ [109], GeO_2_ [112], MoO_3_ [115], SiO_2_ [120], SnO_2_ [123], TiO_2_ [127,133], NiCo_2_O_4_ [138], Li_x_M_y_O_2_ (M = Li, Ni, Co, Mn, Al, …) [144,147,182], Li_4_Ti_5_O_12_ [244,245], Li_3_VO_4_ [260], Na_4_Mn_9_O_18_ [267], LiFePO_4_ [282,292,296,304], LiMnPO_4_ [321], NaTi_2_(PO_4_)_3_ [340], Na_3_V_2_(PO_4_)_3_ [344], Li_3_V_2_(PO_4_)_3_ [325,327], NiS [375], MoS_2_ [353]
Carbon black (CB)	C [33], S [38], LiMnBO_3_ [20], Na_2_FePO_4_F [89], Mn_0.5_Co_0.5_Fe_2_O_4_ [137], Li_4_Ti_5_O_12_ [220,246], LiFePO_4_ [298,302]
Graphite	C [25,26,27,29], Si [43,44,50,52,53,56,58,61,65,66,68,70,71,73,78,79,118], SiO [119]
Others	Carbon (nano)fibers: Si [52], Li_4_Ti_5_O_12_ [234]; Graphitized needle coke: Si [64]; Graphitized carbon black: Si [75]

**Table 6:** Spray-drying in the preparation of Si-carbon composites, starting from Si. For synthesis of Si/C composites starting from SiO_2_, see [48,49]. Unless otherwise stated, Si is “nano” (either purchased as such or ground by ball-milling). CNT = carbon nanotubes; GO = graphene oxide; n.a. = not available.

Reference	Suspension Composition	Post-SD Treatment	%Si
**A. Spray-drying of suspension**			
Li et al. [55]	Hydroxylated Si and carboxylic-functionalized CNT in water	-	70
Wang et al. [69]	Functionalized Si and functionalized CNT in ethanol	-	56 (EDX)
Yang et al. [72]	Si, lithium acetate and ammonium fluoride in ethanol-water	-	94
**B. Spray-drying of suspension followed by heat treatment in inert/reducing atmosphere**			
Bie et al. [42]	Si, CNT and phenol-formaldehyde resin in ethanol	900 °C in Ar	69
Gan et al. [50]	Si and graphite dispersed in GO suspension	600 °C in Ar	10
He et al. [51]	Si in GO suspension	700 °C in Ar/H_2_	81
Lai et al. [53]	Si, graphite, glucose and sodium dodecyl benzene sulfonate in water	800 °C in Ar	25
Lee et al. [54]	Si and GO in aqueous ethanol	700 °C in Ar	63
Liu et al. [61]	Si, graphite and chitosan in water	700 °C in Ar	15
Pan et al. [63]	Si, GO and sucrose	800 °C in Ar/H_2_	72
Su et al. [65]	Si, graphite, phenolic resin and sodium dodecyl benzene sulfonate in water-ethanol	700 °C in Ar	n.a.
Su et al. [66]	Si, graphite and GO in water with 5% alcohol	450 °C in Ar	16
Tao et al. [67]	Si, GO and polyvinyl alcohol in water	700 °C in Ar/H_2_	49
Wang et al. [68]	Si/poly (acrylonitrile-co-divinylbenzene) hybrid microspheres, graphite and sodium carboxymethyl cellulose in water	900 °C in Ar	10
Wang et al. [81]	Micron-sized Si (with SiO_x_ surface layer) and citric acid in water (SiO_x_ not reduced by heat treatment)	600 °C in Ar	85-94
Wang et al. [70]	Microspheres of Si with in situ polymerized styrene-acrylonitrile copolymer, added to a dispersion of graphite and sodium carboxymethyl cellulose in water	900 °C in Ar	6.7
Yang et al. [73]	Si, pitch, CNT and graphite in alcohol-water	850 °C in Ar	30-35
Zhang et al. [75]	Si, graphitized carbon black and sucrose in water	900 °C in N_2_	5-10
Zhang et al. [77]	Si, CNT and glucose in water	800 °C in Ar	n.a.
**C. Two consecutive spray-dryings of suspension with intermediate and final heat treatment in inert/reducing atmosphere**			
Chen et al. [43]	(Step 1) Si, polystyrene-acrylonitrile, citric acid and graphite in ethylene-glycol ; (Step 2) Powder from step 1 mixed with pitch in tetrahydrofuran	(1) 380 °C in N_2_ (2) 500 °C and 900 °C in N_2_	25
Chen et al. [44]	(Step 1) Si, graphite and citric acid in water; (Step 2) Powder from step 1 mixed with pitch in tetrahydrofuran	(1) 380 °C in N_2_ (2) 500 °C and 900 °C in N_2_	6
Chen et al. [45]	(Step 1) Si, graphite and phenol-formaldehyde in ethanol; (Step 2) Powder from step 1 mixed in phenol-formaldehyde solution	(1) and (2) 1000 °C in Ar/H_2_	20
Li et al. [58]	(Step 1) Si, graphite, citric acid, polyvinylpyrrolidone in ethanol; (Step 2) Powder from step 1 mixed with pitch in tetrahydrofuran	(1) 380 °C in N_2_ (2) 500 °C and 900 °C in N_2_	8
**D. Spray-drying of suspension followed by more complex post-processing**			
Li et al. [56]	Si, graphite and glucose in water	Dispersion in pitch solution; drying at 80 °C in vacuum; 1050 °C in Ar; crushing	15
Li et al. [57]	Ball-milled Si in ethanol	HF etching of amorphous SiO_x_ surface layer	100
Li et al. [59]	Si and polyvinyl alcohol in water	Coating with poly-acrylonitrile; 800 °C in Ar	70
Lin et al. [60]	Si and GO in water-ethanol	Reduction and N-doping of GO by hydrazine hydrate vapor	89
Paireau et al. [62]	Si and polyvinyl alcohol in water	PVA crosslinking; 1050 °C in N_2_	40–98
Ren et al. [64]	Si, graphitized needle coke and sucrose in water	900 °C in N_2_; carbon coating by CVD	17
Zhang et al. [74]	Si, NaCl and polyvinyl pyrrolidone in water	900 °C in N_2_; washing of NaCl in water	30
Zhang et al. [76]	Si, polyvinyl pyrrolidone, nickel acetate and citric acid in ethanol (spray-drying in N_2_ atmosphere)	380 °C in N_2_; growth of carbon nanotubes and nanofibers in C_2_H_2_/H_2_ at 700 °C (NiO catalyst)	70
Zhou et al. [78]	Si, graphite and citric acid in alcohol-water	400 °C in Ar; coating in dopamine solution; treatment in Ar at temperatures from 600 to 900 °C	n.a.

## Appendix A

**Table A1:** Spray-drying parameters for layered oxides AMO_2_ (A = Li^+^, Na^+^; M = Li, Ni, Mn, Co, Al, …) Sections in the table are the same as in Table 3 (see main text) where compound stoichiometries and solution/suspension compositions can be found. Information about the spray-drying instruments is given as provided in the referenced papers. - = not available.

	T_inlet_ (°C)	T_outlet_ (°C)	Other Parameters	Spray-Drying Instrument
**SPRAY-DRYING OF SOLUTIONS**				
**A. Spray-drying of aqueous solution of nitrates and/or acetates**				
Duvigneaud et al. [145]	190	150	-	Buchi mini spray-dryer 190
He et al. [146] and He et al. [148]	200	-	400 mL/h Bifluid nozzle 0.2 MPa	SD-2500 (Shanghai Triowin Lab Technology Company)
Kim et al. [152]	-	-	-	-
Kim et al. [187]	-	-	-	SD-1000, Tokyo Rikakikai Co. Ltd, Tokyo, Japan
Konstantinov et al. [153]	190–200	90–100	-	Yamato GA32
Li et al. [154]	-	-	-	Yamato GB32 pulvis mini-spray
Li et al. [156] and Li et al. [157]	-	-	-	Buchi mini spray-dryer B-290
Li et al. [160]	300	100	Bifluid nozzle 0.4 MPa	-
Liu et al. [166]	350	150	10 L/h Bifluid nozzle 0.4 MPa	-
Wang et al. [263]	-	-	-	-
Wang et al. [172]	200	-	2.5 mol/L total cation concentration	-
Wang et al. [173]	210	110	-	-
Wu et al. [175]	220	110	Air pressure 0.2 MPa	-
Yue et al. [179,180]	220	110	-	-
Zhang et al. [183] and Zhang et al. [186] and Zhao et al. [264]	-	-	-	Pulvis mini-spray GB22, Yamato, Japan
**B. Spray-drying of aqueous solution of salts dissolved in aqueous citric acid**				
Li et al. [158]	180	65–70	-	-
Sun et al. [171]			2 mol/L concentration	Pulvis mini-spray GB22, Yamato, Japan
Watanabe et al. [174]	-	-	2 mol/L concentration	Buchi B-290
Zhang et al. [184,185]	-	-	-	Pulvis mini-spray GB22, Yamato, Japan
**C. Spray-drying of aqueous solution of citrates**				
Li et al. [155]	-	-	-	Yamato GB32 pulvis mini-spray
Qiao et al. [169]	-	-	-	L217, Lai Heng
Yuan et al. [178]	-	-	-	L217, Lai Heng
**D. Spray-drying of aqueous solution (others)**				
Li et al. [159]	200	-	Pumping 1.2 g/s Jet-air speed 6 kg/h 4 wt % solution	Spray-dryer Minor Niro A/S, Söborg, Denmark
Oh et al. [167]	-	-	-	-
**SPRAY-DRYING OF SUSPENSIONS**				
**E. Spray-drying of an aqueous suspension to mix reactants**				
Hou et al. [149]	-	-	-	-
Lin et al. [164]	200	-	-	-
Liu et al. [165]	-	-	-	-
Wang et al. [189]	-	-	-	-
Yue et al. [181]	-	-	-	-
**F. Spray-drying of an ethanol suspension to mix reactants**				
Hu et al. [150]	-	-	-	-
Lin et al. [161]	-	-	-	Niro 2108, Copenhagen
Lin et al. [162]	150	-	-	Niro 2108, Copenhagen
**G. Mixing of AMO_2_ active material with conductive carbon or conductive carbon precursor**				
Cheng et al. [144]	200	-	Bifluid nozzle 0.2 MPa	SD-2500
Xia et al. [176]	-	-	-	SD-1500 laboratory scale spray-dryer (Tridwin Tech. Co. Shanghai, China)
Yang et al. [177]	220	-	1.5 L/h Atomization pressure 0.5 MPa	-
Yue et al. [182]	-	-	-	-
**H. Shaping of AMO_2_ as spheres**				
Chen et al. [143]	220	90	Compressed air pressure 0.2 MPa	-

**Table A2:** Inventory of the starting materials used in the publications referenced in this review.

Element	Precursor
Al	Nitrate [145,250,337]
B	H_3_BO_3_ [20], LiBO_2_.8H_2_O[21,22]
Ce	Nitrate [98]
Co	Acetate [146,148,151,152,153,154,155,156,157,160,171,172,173,174,175,177,178,179,180,186,187,200,201,215], nitrate [92,99,100,101,102,138,139,145,166,213,355], Co_3_O_4_ [7,161,162,163,189], Co(OH)_2_ [159,167], (Co,Ni,Mn)OH_x_ [149,165,188], (Co,Ni,Mn)O_x_ [150]
Cr	Acetate [146,203], chloride [103], sulfate [203], Cr_2_O_3_ [202,203]
Cu	Acetate [140,346], nitrate [104,105,106,366]
F	NaF [87,88,89,91,92,93,94], HF [84], NH_4_F [72], trifluoroacetic acid CF_3_COOH [85,86]
Ge	GeO_2_ dissolved in ammonia solution [111], GeO_2_ from hydrolysis of GeCl_4_ [112]
Fe	Fe [87,88,89]
Fe^2+^	Oxalate [271,273,274,275,279,280,317,323,337,349,350,351,352,359], sulfate [135,281,296,304,310], acetate [86,305], chloride [310], (Fe,Mn)_3_(PO_4_)_2_.xH_2_O [312,313,314,319]
Fe^3+^	Nitrate [21,22,110,136,158,212,268,269,270,285,286,300,301,302,303,310,347,354,361,363,366], phosphate [272,277,278,283,284,287,288,290,291,292,293,294,299,306,307,308,311], citrate [107,108,295,360,362], Fe_2_O_3_ [109,190,276],
La	Nitrate [113,357]
Li	Carbonate [7,20,84,149,153,155,161,162,163,164,167,188,189,190,202,203,216,217,218,220,221,227,228,230,231,232,237,243,247,248,251,258,259,260,261,271,273,277,283,286,294,295,299,300,301,302,303,308,317,323,325,329,333,334,362], hydroxide [150,159,165,168,169,172,177,178,184,185,192,193,199,210,222,223,224,225,226,229,233,235,239,240,241,249,253,254,255,256,257,262,272,278,279,281,284,285,287,288,290,291,292,293,296,304,306,311,312,313,314,319,327,331,332,335,336,337,349,351,358,359,360,363], acetate [72,85,86,146,148,160,171,173,174,175,179,180,183,191,194,195,196,197,198,200,201,204,205,208,209,211,212,215,250,274,275,305,307,357], nitrate [145,151,152,154,156,157,158,166,186,187,244,324,364], oxalate [350,352], LiBO_2_.8H_2_O [21,22], LiH_2_PO_4_ [276,280,310,328,365]
Mg	Acetate [308]
Mn	Acetate [90,91,114,140,146,148,151,152,154,155,156,157,168,169,171,172,173,174,177,178,179,180,183,186,187,191,192,193,194,195,196,197,198,200,201,204,205,206,207,208,209,211,212,213,214,215,262,263,264,357,365], nitrate [158,166,199,310,311,364], carbonate [20,161,162,163,189,192], chloride [310], sulfate [310], MnC_2_O_4_.2H_2_O [317,323], MnO_2_ [7,190,202,203], Mn_3_O_4_ [164], (Co,Ni,Mn)OH_x_ [149,165,188], (Co,Ni,Mn)O_x_ [150], (Ni,Mn) oxalate [210], (Fe,Mn)_3_(PO_4_)_2_.xH_2_O [312,313,314,319]
Mo	(NH_4_)_6_Mo_7_O_24_⋅4H_2_O [115,141,367], MoS_2_ [353]
Na	NaOH [87,88,89,97], acetate [263,265,343], Na_2_CO_3_ [339,340,344,346,351], NaHCO_3_ [342], NaNO_3_ [264,347], NaF [87,88,89,90,91,92,93,94], NaH_2_PO_4_ [91,345], sodium carboxymethylcellulose [333]
Ni	Acetate [22,85,117,146,148,151,152,154,155,156,157,168,169,171,172,174,175,177,178,179,180,183,184,185,186,187,204,205,207,208,209,211,212,215,263,264,357,358,375], nitrate [138,139,141,145,158,166,206,213,214,367], carbonate [164], Ni(OH)_2_ [167], NiO [7,161,162,163,189], (Co,Ni,Mn)OH_x_ [149,165,188], (Co,Ni,Mn)O_x_ [150], (Ni,Mn) oxalate [210]
Nb	Nb_2_O_5_ [142,190], (NH_4_)NbO(C_2_O_4_)_2_·H_2_O [131], ethoxide [251]
P	NH_4_H_2_PO_4_ [87,88,89,90,93,94,268,269,270,271,273,274,275,279,285,286,295,301,302,305,317,323,324,325,327,329,332,333,334,335,337,339,340,342,343,344,346,359,360,362], NaH_2_PO_4_ [91,345], LiH_2_PO_4_ [276,280,310,328,365], H_3_PO_4_ [92,93,281,296,300,303,304,312,313,314,319,331,336,363,364], FePO_4_(.xH_2_O) [272,278,283,284,287,288,290,291,292,293,299,306,307,308,351], 1-hydroxyethane 1,1-diphosphonic acid HEDP (CH_3_C(OH)(H_2_PO_3_)_2_) [347,348], P [36], (Fe,Mn)_3_(PO_4_)_2_.xH_2_O [312,313,314,319]
Ru	Acetate [212]
S	Thiourea [114], sulfur [37,38,39], MoS_2_ [353]
Sb	SbCl_3_ [40]
Se	Se [83], H_2_SeO_3_ by dissolving SeO_2_ in water [354], H_2_Se gas for post-treatment of spray-dried precursor [367]
Si	Si [42,43,44,45,46,47,50,51,53,54,55,56,57,58,59,60,62,63,64,65,66,67,69,71,73,74,75,76,77,78,79,80,81,366], SiO_2_ [48,49,120,121,349], SiO [52,118,119], tetraethyl ethoxysilane TEOS [350,352], Si/poly(acrylonitrile-divinylbenzene) hybrid microspheres [68], Si/poly(styrene-acrylonitrile) hybrid microspheres [70]
Sn^2+^	Oxalate [122,124,355], chloride [169]
Sn^4+^	Chloride [82,123,348]
Ti	TiO_2_ [84,133,142,216,217,218,221,223,224,225,226,228,230,231,232,233,237,244,247,308,323,339,340], TiO_2_ from basic hydrolysis of TiOSO_4_·H_2_SO_4_·8H_2_O [126], TiOSO_4_·H_2_SO_4_·H_2_O [131], Ti peroxo-carbonate solution [127], acidic solution of [NH_4_]_2_[Ti(C_2_O_4_)_3_] [184,185], titania nanosheets [129,130], TiO(OH)_2_(·xH_2_O) [220,358], Ti tetraisopropoxide (C_3_H_7_O)_4_Ti [128,132,212,227,235,243,248,251], Ti tetrabutoxide (C_4_H_9_O)_4_Ti [211,222,229,238,239,240,241,249,250,265]
V	NH_4_VO_3_ [94,254,257,324,327,329,331,332,333,335,336,337,342,343,345,346,352,359,361,363,365], V_2_O_5_ [93,253,255,256,258,259,260,261,325,328,334,344,360,362,364],
Zn	Sulfate [135], nitrate [136]
Zr	ZrO_2_ [161], Zr(NO_3_)_4_.5H_2_O [357]

**Table A3:** Discharge specific capacity (in mAh/g) after 50 cycles at the indicated current density (in A/g or as a C-rate). For counter electrode, see first column.

Compound Type, Formulas and References	Discharge Capacity after 50 Cycles	
**Borates**		
LiFeBO_3_ vs. Li [21]	127 mAh/g	after 30 cycles at 10 mA/g + 20 cycles at 20 mA/g
LiFe_0.94_Ni_0.06_BO_3_ vs. Li [22]	132 mAh/g	after 35 cycles at 10 mA/g + 15 cycles at 20 mA/g
**Elements**		
C vs. Li [25]	355 mAh/g	after 50 cycles at 0.1 A/g
C vs. Li [27]	460 mAh/g	after 50 cycles at 0.37 A/g (1 C)
C vs. Li [31]	245 mAh/g	after 50 cycles at 0.1 A/g
C vs. Li [33]	460 mAh/g	after 50 cycles at 0.05 A/g
C (with 4 wt % Ni) vs. Li [35]	640 mAh/g	after 50 cycles at 0.5 A/g
P/C vs. Na [36]	2200 mAh/g	after 50 cycles at 0.1 A/g
S/C vs. Li [37]	980 mAh/g	after 50 cycles at 0.2 C
C/S vs. Li [38]	980 mAh/g	after 50 cycles at 0.1 C
S/C vs. Li [39]	840 mAh/g	after 50 cycles at 0.1 C
Sb/C vs. Na [40]	630 mAh/g	after 50 cycles at 0.2 A/g (0.33 C)
Si/C vs. Li [41]	1150 mAh/g	after 50 cycles at 0.45 A/g
Si/C vs. Li [42]	2200 mAh/g	after 50 cycles at 0.3 A/g
Si/C vs. Li [43]	1150 mAh/g	after 50 cycles at 0.1 A/g
Si/C vs. Li [44]	500 mAh/g	after 50 cycles at 0.1 A/g
Si/C vs. Li [46]	900 mAh/g	after 50 cycles at 0.2 A/g
Si/C vs. Li [47]	2450 mAh/g	after 50 cycles at 0.3 A/g
Si/C vs. Li [48]	1100 mAh/g	after 50 cycles at 0.3 A/g
Si/C vs. Li [49]	2200 mAh/g	after 50 cycles at 1 A/g
Si/C vs. Li [50]	420 mAh/g	after 50 cycles at 0.05 A/g
Si/C vs. Li [52]	600 mAh/g	after 50 cycles at 0.1 A/g
Si/C vs. Li [54]	1250 mAh/g	after 50 cycles at 1 A/g
Si/C vs. Li [55]	2100 mAh/g	after 50 cycles at 0.5 C
Si/C vs. Li [56]	570 mAh/g	after 50 cycles at 0.1 C
Si/C vs. Li [58]	650 mAh/g	after 50 cycles at 0.1 A/g
Si/C vs. Li [60]	1160 mAh/g	after 50 cycles at 0.1 A/g
Si/C vs. Li [61]	580 mAh/g	after 50 cycles at 0.1 A/g
Si/C vs. Li [63]	1800 mAh/g	after 50 cycles at 0.2 A/g
Si/C vs. Li [64]	560 mAh/g	after 50 cycles at 0.05 A/g
Si/C vs. Li [65]	500 mAh/g	after 50 cycles at 0.1 A/g
Si/C vs. Li [66]	500 mAh/g	after 50 cycles at 0.1 A/g
Si/C vs. Li [67]	950 mAh/g	after 50 cycles at 0.1 A/g
Si/C vs. Li [68]	500 mAh/g	after 50 cycles at 0.1 A/g
Si/C vs. Li [69]	2100 mAh/g	after 50 cycles at 0.5 A/g
Si/C vs. Li [70]	450 mAh/g	after 50 cycles at 0.1 A/g
Si/C vs. Li [71]	500 mAh/g	after 50 cycles at 5 C
Si/C vs. Li [73]	820 mAh/g	after 50 cycles at 0.1 A/g
Si/C vs. Li [74]	1400 mAh/g	after 50 cycles at 0.05 C
Si/C vs. Li [75]	500 mAh/g	after 50 cycles at 0.05 A/g
Si/C vs. Li [76]	1200 mAh/g	after 50 cycles at 0.3 A/g
Si/C vs. Li [77]	1100 mAh/g	after 50 cycles at 0.2 A/g
Si/C vs. Li [78]	780 mAh/g	after 50 cycles at 0.2 A/g
Si/C vs. Li [79]	1700 mAh/g	after 50 cycles at 1 C
Si/C vs. Li [80]	1550 mAh/g	after 50 cycles at 0.05 A/g
Si/C vs. Li [81]	1860 mAh/g	after 50 cycles at 0.1 A/g
Sn/C vs. Li [82]	670 mAh/g	after 50 cycles at 0.2 A/g
Sn/C vs. Na [82]	400 mAh/g	after 50 cycles at 0.05 A/g
Se/C vs. Li [83]	590 mAh/g	after 50 cycles at 0.1 C
**Fluorides**		
Li_3_FeF_6_ vs. Li [86]	85 mAh/g	after 50 cycles at 0.05 C
**Fluorophosphates**		
Na_2_MnPO_4_F/C vs. Na [90]	77 mAh/g	after 50 cycles at 6.2 mA/g
Na_3_V_2_(PO_4_)_2_F_3_/C vs. Li [93]	100 mAh/g	after 50 cycles at 1 C
Na_3_V_2_O_2_(PO_4_)_2_F/C vs. Na [94]	117 mAh/g	after 50 cycles at 0.5 C
**Organic salts**		
Li_2_C_8_H_4_O_4_ vs. Li [95]	150 mAh/g	after 50 cycles at 0.05 C
Na_2_C_8_H_4_O_4_/C vs. Li [96]	210 mAh/g	after 50 cycles at 0.1 C
**Oxides M_x_O_y_**		
CoO/C vs. Li [100]	900 mAh/g	after 50 cycles at 1.4 A/g
Co_3_O_4_ vs. Li [100]	830 mAh/g	after 50 cycles at 1.4 A/g
Co_3_O_4_ vs. Li [101]	1020 mAh/g	after 50 cycles at 0.5 A/g
Co_3_O_4_ vs. Li [102]	1050 mAh/g	after 50 cycles at 1.4 A/g
Cr_2_O_3_/C vs. Li [103]	630 mAh/g	after 50 cycles at 0.1 A/g
CuO vs. Li [104]	690 mAh/g	after 50 cycles at 1 A/g
CuO/C vs. Li [105]	700 mAh/g	after 50 cycles at 2 A/g
CuO vs. Li [106]	760 mAh/g	after 50 cycles at 1 A/g
Fe_2_O_3_ vs. Li [107]	870 mAh/g	after 50 cycles at 0.4 A/g
Fe_2_O_3_/C vs. Li [108]	880 mAh/g	after 50 cycles at 0.4 A/g
Fe_2_O_3_/C vs. Li [109]	710 mAh/g	after 50 cycles at 0.8 A/g
Fe_2_O_3_ vs. Li [110]	1020 mAh/g	after 50 cycles at 0.4 A/g
GeO_x_/C vs. Li [111]	975 mAh/g	after 50 cycles at 0.5 A/g
GeO_2_/C vs. Li [112]	1060 mAh/g	after 50 cycles at 0.2 C
MnO/C vs. Li [114]	300 mAh/g	after 50 cycles at 0.5 A/g
MoO_3_/C vs. Li [115]	1120 mAh/g	after 50 cycles at 0.5 A/g
NiO vs. Li [117]	590 mAh/g	after 50 cycles at 0.1 C
SnO_2_/C vs. Li [122]	600 mAh/g	after 50 cycles at 2 A/g
SnO_2_/C vs. Li [123]	1200 mAh/g	after 50 cycles at 0.1 A/g
SnO_2_ vs. Li [124]	715 mAh/g	after 50 cycles at 2 A/g
SnO_2_ vs. LiMn_2_O_4_ [124]	365 mAh/g	after 50 cycles at 1 A/g
TiO_2_ vs. Li [126]	75 mAh/g	after 50 cycles from 0.1 C to 10 C
TiO_2_/C vs. Li [127]	150 mAh/g	after 50 cycles at 0.94 A/g
TiO_2_ vs. Li [130]	80 mAh/g	after 50 cycles at 0.02A/g
TiO_2_ vs. Li [131]	190 mAh/g	after 50 cycles at 0.5 C
TiO_2_/C vs. Na [133]	140 mAh/g	after 50 cycles at 0.2 C
V_2_O_5_/C vs. Li [134]	240 mAh/g	after 50 cycles at 0.2 C
**Oxides M_x_M’_y_O_z_**		
ZnFe_2_O_4_ vs. Li [135]	1250 mAh/g	after 50 cycles at 0.1 A/g
ZnFe_2_O_4_ vs. Li [136]	750 mAh/g	after 50 cycles at 0.5 A/g
Mn_0.5_Co_0.5_Fe_2_O_4_/C vs. Li [137]	610 mAh/g	after 50 cycles at 0.1 A/g
(Ni,Co)O_x_ vs. Li [139]	850 mAh/g	after 50 cycles at 1 A/g
Cu_1.5_Mn_1.5_O_4_ vs. Li [140]	460 mAh/g	after 50 cycles at 0.1 A/g
NiMoO_4_ vs. Li [141]	1000 mAh/g	after 50 cycles at 1 A/g
TiNb_2_O_7_/C vs. Li [142]	300 mAh/g	after 50 cycles at 0.25 C
**Oxides Li_x_M_y_O_z_ (layered)**		
LiCoO_2_ vs. graphite [153]	132 mAh/g	after 50 cycles at 0.3 mA/g
LiNi_0.8_Co_0.2_O_2_ vs. Li [167]	160 mAh/g	after 50 cycles at 0.5 C
LiNi_0.8_Co_0.15_Al_0.05_O_2_ vs. Li [143]	151 mAh/g	after 50 cycles at 2 C
LiNi_0.6_Co_0.2_Mn_0.2_O_2_ vs. Li [179]	132 mAh/g at 50 °C	after 50 cycles at 0.16 A/g
LiNi_0.6_Co_0.2_Mn_0.2_O_2_ vs. Li [180]	135 mAh/g	after 50 cycles at 0.08 A/g
LiNi_0.6_Co_0.2_Mn_0.2_O_2_/C vs. Li [182]	154 mAh/g	after 50 cycles at 0.5 C
LiNi_0*.*425_Mn_0*.*425_Co_0*.*15_O_2_ vs. Li [155]	110 mAh/g	after 50 cycles at 1 C
LiMn_1/3_Ni_1/3_Co_1/3_O_2_ (ZrO_2_-coated) vs. Li [156]	140 mAh/g	after 50 cycles at 0.5 C
LiMn_1/3_Ni_1/3_Co_1/3_O_2_-0.1 LiF vs. Li [157]	133 mAh/g	after 50 cycles at 0.32 A/g
LiMn_1/3_Ni_1/3_Co_1/3_O_2_ vs. Li [163]	180 mAh/g	after 50 cycles at 0.2 C
LiMn_1/3_Ni_1/3_Co_1/3_O_2_ vs. Li [165]	160 mAh/g	after 50 cycles at 1 C
0.98 LiCoO_2_-0.02 Li_2_MnO_3_ vs. Li [173]	140 mAh/g	after 50 cycles at 1 C
Li_1.06_Ni_0.3_Co_0.4_Mn_0.3_O_2-d_ vs. Li [187]	180 mAh/g	after 50 cycles at 0.03 A/g
Li_1.11_(Ni_0.4_Co_0.2_Mn_0.4_)_0.89_O_2_ vs. Li [152]	187 mAh/g at 50 °C	after 50 cycles at 0.1 A/g
0.7 LiMn_0.337_Ni_0.487_Co_0.137_Cr_0.04_O_2_ -0.3 Li_2_MnO_3_ vs. Li [146]	158 mAh/g	after 20 cycles at 0.05 A/g + 30 cycles at 0.25 A/g
0.7 LiMn_0.5_Ni_0.4_Co_0.1_O_2_ -0.3 Li_2_MnO_3_ vs. Li [148]	200 mAh/g	after 50 cycles at 0.05 A/g (0.2 C)
Li_1.17_(Mn_1/3_Ni_1/3_Co_1/3_)_0.83_O_2_ vs. Li [151]	177 mAh/g	after 50 cycles at 0.03 A/g
Li_1.17_Ni_0.2_Co_0.05_Mn_0.58_O_2_ (CeO_2_-coated) vs. Li [178]	212 mAh/g	after 50 cycles at 0.3 A/g
Li_1.17_Ni_0.25_Mn_0.58_O_2_ (Li-Mn-PO_4_-coated) vs. Li [168]	265 mAh/g	after 50 cycles at 0.03 A/g
Li_1.17_Ni_0.25_Mn_0.55_Sn_0.03_O_2_ vs. Li [169]	170 mAh/g	after 50 cycles at 0.3 A/g
Li_1.2_Mn_0.54_Co_0.13_Ni_0.13_O_2_/C vs. Li [144]	160 mAh/g	after 20 cycles at 0.2 C + 30 cycles at 1 C
Li_1.2_Mn_0.54_Ni_0.13_Co_0.13_O_2_/C vs. Li [147]	177 mAh/g	after 20 cycles at 0.05 A/g + 30 cycles at 0.125 A/g
Li_1.2_Ni_0.13_Co_0.13_Mn_0.54_O_2_ vs. Li [188]	160 mAh/g	after 50 cycles from 0.1 C to 0.5 C
Li_1.2_Mn_0.54_Ni_0.13_Co_0.13_O_2_ vs. Li [189]	200 mAh/g	after 50 cycles at 1 C
Li_1.2_Ni_0.13_Co_0.13_Mn_0.54_O_2_/C vs. Li [177]	175 mAh/g	after 50 cycles from 0.2 C to 5 C
Li_1.2_Ni_0.2_Mn_0.6_O_2_ vs. Li [164]	150 mAh/g	after 50 cycles at 0.5 C
0.5 LiMn_1/3_Ni_1/3_Co_1/3_O_2_ -0.5 Li_2_MnO_3_ vs. Li [149]	189 mAh/g	after 50 cycles at 1 C
0.5 LiMn_1/3_Ni_1/3_Co_1/3_O_2_ -0.5 Li_2_MnO_3_ vs. soft C [172]	190 mAh/g	after 50 cycles at 1 C
0.95 LiNiO_2_-0.05 Li_2_TiO_3_ vs. Li [184]	175 mAh/g	after 50 cycles at 0.02 A/g
**Oxides Li_x_M_y_O_z_ (others)**		
LiMn_2_O_4_ vs. Li [191]	113 mAh/g	after 50 cycles at 1 C
LiMn_2_O_4_ vs. Li [192]	117 mAh/g	after 50 cycles at 0.2 C
LiMn_2_O_4_ vs. Li [193]	110 mAh/g	after 50 cycles at 0.2 C
LiMn_2_O_4_ vs. Li [194]	113 mAh/g	after 50 cycles at 1 C
LiMn_2_O_4_ vs. Li [198]	113 mAh/g	after 50 cycles at 1 C
LiMn_2_O_4_ vs. Li [199]	106 mAh/g	after 50 cycles at 0.5 C
LiMn_11/6_Co_1/6_O_4_ vs. Li [201]	112 mAh/g	after 50 cycles at 0.2 C
LiNi_0.5_Mn_1.5_O_4_ vs. Li [206]	135 mAh/g	after 50 cycles at 0.15 C
LiNi_0.5_Mn_1.5_O_4_ vs. Li [207]	132 mAh/g	after 50 cycles at 0.1 C
LiNi_0.5_Mn_1.5_O_4_ vs. Li [208]	118 mAh/g	after 50 cycles at 2 C
LiNi_0.5_Mn_1.5_O_4_/C vs. Li [210]	130 mAh/g	after 50 cycles at 0.5 C
LiNi_0.5_Mn_1.47_Ti_0.03_O_4_ vs. Li [211]	125 mAh/g	after 50 cycles from 0.05 C to 5 C
LiNi_0.5_Mn_1.4_Fe_0.1_Ti_0.03_O_4_ vs. Li [212]	170 mAh/g	after 50 cycles at 0.5 C
LiNi_0.5_Mn_1.4_Ru_0.1_Ti_0.03_O_4_ vs. Li [212]	180 mAh/g	after 50 cycles at 0.5 C
LiNi_0.3_Mn_1.5_Co_0.2_O_4_ vs. Li [213]	115 mAh/g at 60 °C	after 50 cycles at 3.5 C
LiNi_0.45_Mn_1.5_Co_0.05_O_4_ vs. Li [214]	126 mAh/g	after 50 cycles at 0.15 C
Li_4_Ti_5_O_12_ vs. Li [216]	147 mAh/g at 50 °C	after 50 cycles at 1 C
Li_4_Ti_5_O_12_ vs. Li [217]	150 mAh/g	after 50 cycles at 1 C
Li_4_Ti_5_O_12_/C vs. Li [219]	150 mAh/g	after 50 cycles at 2 C
Li_4_Ti_5_O_12_ vs. Li [220]	150 mAh/g	after 50 cycles at 1 C
Li_4_Ti_5_O_12_ vs. Li [222]	160 mAh/g	after 50 cycles at 1 C
Li_4_Ti_5_O_12_ vs. Li [223]	175 mAh/g	after 50 cycles at 0.2 C
Li_4_Ti_5_O_12_/C vs. Li [226]	165 mAh/g	after 50 cycles at 1 C
Li_4_Ti_5_O_12_ vs. Li [229]	211 mAh/g	after 50 cycles at 2 C
Li_4_Ti_5_O_12_/C vs. Li [230]	155 mAh/g	after 50 cycles at 1 C
Li_4_Ti_5_O_12_ vs. Li [233]	162 mAh/g	after 50 cycles at 1 C
Li_4_Ti_5_O_12_ vs. Li [234]	170 mAh/g	after 50 cycles at 1 C
Li_4_Ti_5_O_12_/C vs. Li [235]	164 mAh/g	after 50 cycles at 1 C
Li_4_Ti_5_O_12_/TiO_2_ vs. Li [236]	168 mAh/g	after 50 cycles at 1 C
Li_4_Ti_5_O_12_ vs. Li [239]	168 mAh/g	after 50 cycles at 1 C
Li_4_Ti_5_O_12_ vs. Li [240]	172 mAh/g	after 50 cycles at 1 C
Li_4_Ti_5_O_12_/C vs. Li [241]	142 mAh/g	after 50 cycles at 10 C
Li_4.3_Ti_5_O_12_/C vs. Li [242]	132 mAh/g	after 50 cycles at 3 C
Li_4.3_Ti_5_O_12_ vs. Li [243]	140 mAh/g	after 50 cycles at 1 C
Li_4_Ti_5_O_12_/C vs. Li [245]	158 mAh/g	after 50 cycles at 5 C
Li_4_Ti_5_O_12_/C vs. Li [246]	167 mAh/g	after 50 cycles at 0.1 C
Li_4_Ti_5_O_12_/C vs. Li [247]	143 mAh/g	after 50 cycles at 1 C
Li_4_Ti_5_O_12_/C vs. Li [248]	146 mAh/g	after 50 cycles at 2 C
Li_4_Ti_5_O_12_ vs. Li [249]	168 mAh/g	after 50 cycles at 1 C
Li_3.98_Al_0.06_Ti_4.96_O_12_/C vs. Li [250]	160 mAh/g	after 50 cycles at 1 C
Li_1.1_V_3_O_8_/C vs. Li [254]	225 mAh/g	after 50 cycles at 0.33 C
LiV_3_O_8_ vs. Li [255]	260 mAh/g	after 50 cycles at 0.125 A/g
Li_3_VO_4_/C vs. Li [258]	315 mAh/g	after 50 cycles at 10 C
Li_3_VO_4_/C vs. Li [259]	400 mAh/g	after 50 cycles at 0.2 C
Li_3_VO_4_/C vs. Li [260]	395 mAh/g	after 50 cycles at 0.5 C
Li_4_Mn_5_O_12_ vs. Li [262]	128 mAh/g	after 50 cycles at 0.5 C
**Oxides Na_x_M_y_O_z_**		
Na_2/3_Ni_1/3_Mn_2/3_O_2_ vs. Na [263]	102 mAh/g	after 50 cycles at 0.1 C
Na_2_Ti_3_O_7_ vs. Na [265]	95 mAh/g	after 50 cycles from 0.1 C to 5 C
Na_4_Mn_9_O_18_/C in aqueous Na-ion battery [266]	85 mAh/g	after 50 cycles at 4 C
Na_4_Mn_9_O_18_/C in aqueous Na-ion battery [267]	50 mAh/g	after 50 cycles at 4 C
**Phosphates**		
LiFePO_4_/C vs. Li [271]	159 mAh/g	after 50 cycles at 1 C
LiFePO_4_/C vs. Li [273]	156 mAh/g	after 50 cycles at 1 C
LiFePO_4_/C vs. Li [275]	137 mAh/g	after 50 cycles at 1 C
LiFePO_4_/C vs. Li [276]	110 mAh/g	after 50 cycles at 1 C
LiFePO_4_/C vs. Li [278]	154 mAh/g	after 50 cycles at 1 C
LiFePO_4_/C vs. Li [281]	160 mAh/g	after 50 cycles at 0.1 C
LiFePO_4_/C vs. Li [282]	150 mAh/g	after 50 cycles at 1 C
LiFePO_4_/C vs. Li [283]	160 mAh/g	after 50 cycles at 1 C
LiFePO_4_/C vs. Li [284]	159 mAh/g	after 50 cycles at 0.1 C
LiFePO_4_/C vs. Li [285]	130 mAh/g	after 50 cycles at 5 C
LiFePO_4_/C vs. Li [286]	110 mAh/g	after 50 cycles from 0.1 C to 2 C
LiFePO_4_/C vs. Li [289]	110 mAh/g	after 50 cycles at 10 C
LiFePO_4_/C vs. Li [290]	123 mAh/g	after 50 cycles at 10 C
LiFePO_4_/C vs. Li [291]	162 mAh/g	after 50 cycles at 0.5 C
LiFePO_4_/C vs. Li [292]	156 mAh/g	after 50 cycles at 1 C
LiFePO_4_/C vs. Li [293]	120 mAh/g	after 50 cycles at 10 C
LiFePO_4_/C vs. Li [294]	140 mAh/g	after 50 cycles at 2 C
LiFePO_4_/C vs. Li [295]	137 mAh/g	after 50 cycles from 0.1 C to 4 C
LiFePO_4_/C vs. Li [296]	149 mAh/g	after 50 cycles at 1 C
LiFePO_4_/C vs. Li [298]	100 mAh/g	after 50 cycles at 3 C
LiFePO_4_/C vs. Li [299]	147 mAh/g	after 50 cycles at 3 C
LiFePO_4_/C vs. Li [300]	142 mAh/g	after 50 cycles at 0.1 C
LiFePO_4_/C vs. Li [304]	110 mAh/g	after 50 cycles at 10 C
LiFePO_4_/C vs. Li [305]	110 mAh/g	after 50 cycles at 10 C
LiFePO_4_/C vs. Li [306]	120 mAh/g	after 50 cycles at 10 C
LiFePO_4_/C vs. Li [307]	137 mAh/g	after 50 cycles at 1 C
LiFePO_4_/C vs. Li [308]	152 mAh/g	after 50 cycles at 1 C
LiFePO_4_/C vs. Li [309]	105 mAh/g	after 50 cycles at 1 C
LiFe_0.6_Mn_0.4_PO_4_/C vs. Li [315]	137 mAh/g	after 50 cycles at 2 C
LiFe_0.6_Mn_0.4_PO_4_/C vs. Li [316]	150 mAh/g	after 50 cycles at 0.5 C
LiMn_0.5_Fe_0.5_PO_4_/C vs. Li [318]	150 mAh/g at 55 °C	after 50 cycles at 1 C
LiMn_0.6_Fe_0.4_PO_4_/C vs. Li [312]	425 Wh/kg	after 50 cycles at 10 C
LiMn_0.7_Fe_0.3_PO_4_/C vs. Li [319]	145 mAh/g	after 50 cycles at 5 C
LiMn_0.75_Fe_0.25_PO_4_/C vs. Li [310]	120 mAh/g	after 50 cycles at 10 C
LiMn_0.8_Fe_0.2_PO_4_/C vs. Li [313]	138 mAh/g	after 50 cycles at 5 C
LiMn_0.8_Fe_0.2_PO_4_/C vs. Li_4_Ti_5_O_12_ [313]	122 mAh/g	after 50 cycles at 1 C
LiMn_0.8_Fe_0.2_PO_4_/C vs. Li [314]	132 mAh/g	after 50 cycles at 5 C
LiMn_0.85_Fe_0.15_PO_4_/C vs. Li [317]	136 mAh/g	after 50 cycles at 1 C
LiMn_0.85_Fe_0.15_PO_4_/C vs. Li [320]	136 mAh/g	after 50 cycles at 1 C
Li(Mn_0.85_Fe_0.15_)_0.92_Ti_0.08_PO_4_/C vs. Li [323]	144 mAh/g	after 50 cycles at 1 C
LiMn_0.97_Fe_0.03_PO_4_/C vs. Li [311]	158 mAh/g	after 50 cycles at 0.5 C
LiMnPO_4_/C vs. Li [321]	96 mAh/g	after 50 cycles at 0.05 C
LiVOPO_4_ vs. Li [324]	50 mAh/g	after 50 cycles at 0.2 C
Li_3_V_2_(PO_4_)_3_/C vs. Li [325]	143 mAh/g	after 50 cycles at 20 C
Li_3_V_2_(PO_4_)_3_/C vs. Li [326]	100 mAh/g	after 50 cycles from 0.2 C to 20 C
Li_3_V_2_(PO_4_)_3_/C vs. Li [327]	127 mAh/g	after 50 cycles at 0.1 C
Li_3_V_2_(PO_4_)_3_/C vs. Li [328]	131 mAh/g	after 50 cycles at 0.02 A/g
Li_3_V_2_(PO_4_)_3_/C vs. Li [329]	149 mAh/g	after 50 cycles at 10 C
Li_3_V_2_(PO_4_)_3_/C vs. Li [330]	118 mAh/g	after 50 cycles from 0.1 C to 5 C
Li_3_V_2_(PO_4_)_3_/C vs. Li [332]	123 mAh/g	after 50 cycles at 2 C
Li_3_V_2_(PO_4_)_3_/C vs. Li [333]	131 mAh/g	after 50 cycles at 0.1 C
Li_3_V_2_(PO_4_)_3_/C vs. Li [334]	138 mAh/g	after 50 cycles at 1 C
Li_3_V_2_(PO_4_)_3_/C vs. Li [335]	94 mAh/g	after 50 cycles at 1 C
NaTi_2_(PO_4_)_3_/C vs. Na [339]	110 mAh/g	after 50 cycles from 0.2 C to 4 C
NaTi_2_(PO_4_)_3_/C vs. Na [340]	128 mAh/g	after 50 cycles from 0.1 C to 5 C
NaTi_2_(PO_4_)_3_/C vs. Na_3_V_2_(PO_4_)_3_/C [340]	98 mAh/g	after 50 cycles at 10 C
Na_3_V_2_(PO_4_)_3_/C vs. Na [342]	92 mAh/g	after 50 cycles at 10 C
Na_3_V_2_(PO_4_)_3_/C vs. Na [344]	103 mAh/g	after 50 cycles at 5 C
Na_3_V_2_(PO_4_)_3_/C vs. Na [345]	93 mAh/g	after 50 cycles at 5 C
Na_3_V_1.95_Cu_0.05_(PO_4_)_3_/C vs. Na [346]	103 mAh/g	after 50 cycles at 20 C
**Pyrophosphates**		
Na_2_FeP_2_O_7_/C vs. Na [347]	87 mAh/g	after 50 cycles at 0.1 C
Na_2_FeP_2_O_7_/C vs. hard carbon [347]	62 mAh/g	after 50 cycles at 1 C
SnP_2_O_7_/C vs. Li [348]	645 mAh/g	after 50 cycles at 0.1 C
**Silicates**		
Li_2_FeSiO_4_/C vs. Li [349]	137 mAh/g	after 50 cycles at 1 C
Li_2_FeSiO_4_/C vs. Li [350]	140 mAh/g	after 50 cycles at 0.1 C
Li_1.95_Na_0.05_FeSiO_4_/C vs. Li [351]	138 mAh/g	after 50 cycles at 2 C
Li_2_Fe_0.5_V_0.5_SiO_4_/C vs. Li [352]	157 mAh/g	after 50 cycles at 0.5 C
**Sulfides and selenides**		
MoS_2_/C vs. Li [353]	800 mAh/g	after 50 cycles at 0.1 A/g
MoS_2_/C vs. Na [353]	350 mAh/g	after 50 cycles at 0.1 A/g
FeSe_2_/C vs. Na [354]	510 mAh/g	after 50 cycles at 0.5 A/g
MnS/C vs. Li [114]	700 mAh/g	after 50 cycles at 0.5 A/g
NiS/C vs. Na [375]	490 mAh/g	after 50 cycles at 0.3 A/g
**Composites (not with carbon)**		
Sn–Sn_2_Co_3_@CoSnO_3_–Co_3_O_4_ vs. Li [355]	1050 mAh/g	after 50 cycles at 1 A/g
0.5 LiNi_0.5_Mn_1.5_O_4_-0.5 Li_7_La_3_Zr_2_O_12_ vs. Li [357]	116 mAh/g	after 50 cycles at 1 C
3Li_4_Ti_5_O_12_.NiO [358]	240 mAh/g	after 50 cycles at 1 C
9 LiFePO_4_-1 Li_3_V_2_(PO_4_)_3_/C vs. Li [362]	154 mAh/g	after 50 cycles at 1 C
3 LiFePO_4_-1 Li_3_V_2_(PO_4_)_3_/C vs. Li [360]	152 mAh/g	after 50 cycles at 1 C
0.7 LiFePO_4_ -0.3 Li_3_V_2_(PO_4_)_3_/C vs. Li [359]	120 mAh/g	after 50 cycles from 0.03 A/g to 1.5 A/g
2 LiFePO_4_-1 Li_3_V_2_(PO_4_)_3_/C vs. Li [361]	143 mAh/g	after 50 cycles at 0.1 C
1 LiMnPO_4_-1 Li_3_V_2_(PO_4_)_3_/C vs. Li [364]	123 mAh/g	after 50 cycles at 0.1 C
1 LiMnPO_4_-2 Li_3_V_2_(PO_4_)_3_/C vs. Li [365]	130 mAh/g	after 50 cycles at 0.1 C
Si-FeSi_2_-Cu_3.17_Si vs. Li [366]	410 mAh/g	after 50 cycles at 0.5 C
MoS_2_–Ni_9_S_8_ vs. Na [367]	500 mAh/g	after 50 cycles at 0.5 A/g
MoSe_2_-NiSe-C vs. Na [367]	390 mAh/g	after 50 cycles at 0.5 A/g
